# Differential gene expression between viruliferous and non-viruliferous *Schizaphis graminum* (Rondani)

**DOI:** 10.1371/journal.pone.0294013

**Published:** 2023-11-08

**Authors:** Yan M. Crane, Charles F. Crane, Brandon J. Schemerhorn

**Affiliations:** 1 Crop Production and Pest Control Research Unit, Agricultural Research Service, United States Department of Agriculture, West Lafayette, Indiana, United States of America; 2 Department of Entomology, Purdue University, West Lafayette, Indiana, United States of America; 3 Department of Botany and Plant Pathology, Purdue University, West Lafayette, Indiana, United States of America; Fort Valley State University, UNITED STATES

## Abstract

An experiment was performed to measure the effect of Cereal Yellow-Dwarf Virus (CYDV), strain CYDV-RPV, on gene expression in its insect vector, greenbug aphid (*Schizaphis graminum* (Rondani)). RNA was sampled in three replicates from four treatments (biotypes B and H with or without carried CYDV), at 0, 1, 2, 3, 5, 10, 15 and 20 days from the introduction of carrier and virus-free greenbugs to uninfected wheat cv. ‘Newton’. Illumina paired-end sequencing produced 1,840,820,000,000 raw reads that yielded 1,089,950,000 clean reads, which were aligned to two greenbug, Trinity transcriptome assemblies with bowtie2. Read counts to contigs were analyzed with principal components and with DESeq2 after removing contaminating contigs of wheat or microbial origin. Likelihood ratio tests with one transcriptome showed that CYDV influenced gene expression about seven-fold less than time or biotype, which were approximately equal. With the other transcriptome, virus, time, and biotype were about equally important. Pairwise comparisons of virus to no virus for each timepoint yielded estimates of fold-change that comprised expression profiles for each contig when ordered by timepoint. Hierarchical clustering separated expression profiles into 20 groups of contigs that were significantly differentially expressed for at least one timepoint. Contigs were also sorted by timepoint of maximally differential expression between virus and no virus. All contigs that were significantly differentially expressed at FDR = 0.05 were annotated by blast searches against NCBI nr and nt databases. Interesting examples of up-regulation with virus included a lysosomal-trafficking regulator, peptidylprolylisomerase, RNA helicase, and two secreted effector proteins. However, carried virus did not consistently change aphid gene expression overall. Instead there was complex interaction of time, biotype, host response, and virus.

## Introduction

Greenbug (*Schizaphis graminum* (Rondani)) (Hemiptera: Aphidae) is an economically important vector of yellow dwarf viral diseases in grasses [1, 2], especially small grains. Greenbug feeding and viral transmission were estimated to cost wheat and sorghum growers more than $250 million per year in the US Great Plains [3], and aphid-transmitted viral diseases preclude oat production in the southeastern United States. Barley yellow dwarf (BYD) is a serious disease of cereal crops caused by either of two luteoviruses: barley yellow dwarf virus (BYDV, strain PAV) and cereal yellow dwarf virus (CYDV, strain RPV) [4]. When greenbugs feed on infected wheat, the virus can move from the aphid hindgut through the hemolymph to the salivary glands without replicating or expressing viral genes in the aphid [5]. Intact luteoviral capsids are enclosed within lysosomes as they pass through the gut lining and the salivary gland [5]. Upon feeding, the virus is injected with the saliva into the plant, and the plant becomes infected [5]. It is difficult to deconvolve the effect of the carried virus itself and the altered nutritional content of the infected plant on aphid behavior, but fecundity is decreased in greenbugs feeding on wheat infected with wheat streak mosaic virus [6], and evidence suggests that several plant viruses can affect feeding preference of insect vectors [7].

At least six greenbug biotypes (genetic variants) have been recognized on wheat in the United States [2, 8]. The biotypes are defined by a gene-for-gene interaction with various resistance genes in wheat lines [8]. CYDV strain RPV and greenbug biotypes B and H were used in the experiments presented here.

Currently, RNAseq with next-generation sequencing is a standard way to investigate gene expression over an entire genome. RNAseq provides counts of reads, thus mRNA molecules, for each expressed gene. Software pipelines exist to manipulate counts, infer changes in expression between treatments, and infer networks of expression that might elucidate mechanisms of physiological response. We have used RNAseq to investigate changes in greenbug gene expression over time on CYDV-infected and uninfected wheat.

An RNAseq study in *Schizaphis graminum* [9] found 2782 genes that responded to an insecticide, imidacloprid, including 20 strongly upregulated genes whose products could detoxify imidacloprid. These 20 genes were further studied with real-time PCR and RNAi experiments; the standards for the real-time PCR were chosen as described by Zhang et al. [10]. The transcriptome assembly was annotated and published later with real-time PCR results for 12 additional genes related to imidacloprid detoxification [11], which confirmed their up-regulation and distribution within the aphid body and between life stages. Also, gene expression has been compared across instar stages in alate and apterous forms of the bird cherry-oat aphid, *Rhopalosiphum padi* (L.), using a Trinity assembly of the reads themselves in lieu of a reference genome [12]. That study condensed the Trinity assembly to unigenes before mapping reads and considered in depth 54 differentially expressed genes or proteins with previously known developmental roles. Marmonier et al. [13] studied differential gene expression in *Myzus persicae* (Sulzer) that had or had not acquired turnip yellow virus (TuYV) from *Arabidopsis* plants or an artificial medium. They found that somewhat more genes were differentially expressed on the artificial diet than on plants (201 versus 164 with only four in common) and that aphids moved more after feeding on the artificial medium. A genetic study [14] correlated efficient transmission of CYDV-RPV with an allele at cyclophilin B in a small, segregating F_2_ population of *S*. *graminum*. They also demonstrated binding of the cyclophilin A and B proteins with CYDV-RPV virions and concluded that the efficient allele facilitated movement of virions across the aphid hindgut.

The haploid DNA content of *S*. *graminum* has been measured as c = 0.53 pg [15] to 0.56 pg [16], corresponding to a genome size of 518 to 548 megabases by the conversion formula of 1.00 pg = 978 megabases [17]. An unannotated, scaffold-level, reference genome assembly of *S*. *graminum* has been deposited in GenBank as accession GCA_003264975.1 [18]. Its ungapped length of 359.7 megabases is about 67% of the total genome size. It consists of 7859 scaffolds with an N50 of 1,292,312 bases.

Barley yellow-dwarf virus increases the amino acid content of infected phloem [19], and this improved nutrition affects gene expression in *R*. *padi*, resulting in increased fecundity on infected wheat [20]. Cereal yellow-dwarf virus plausibly would have a similar effect. At the same time, the greenbug immune system must encounter the CYDV virions, since they pass through the gut wall in lysosomes and circulate in the hemolymph on their way to the salivary glands. One way to reveal the effects of nutrition and wheat defense on greenbug gene expression is to move aviruliferous and viruliferous greenbugs to uninfected wheat and follow their response over a time course. During the first days on the initially uninfected wheat, the effect of carried CYDV expectedly sets the most transcriptional difference between viruliferous and aviruliferous greenbugs. The conventional belief is that CYDV does not affect the carrier aphid, at least in any easily measured way. With RNAseq it is possible to discern subtle or invisible changes in carrier greenbugs and to garner some information about the interaction between greenbug and virus.

## Materials and methods

### Greenbug populations

**Fig 1:**
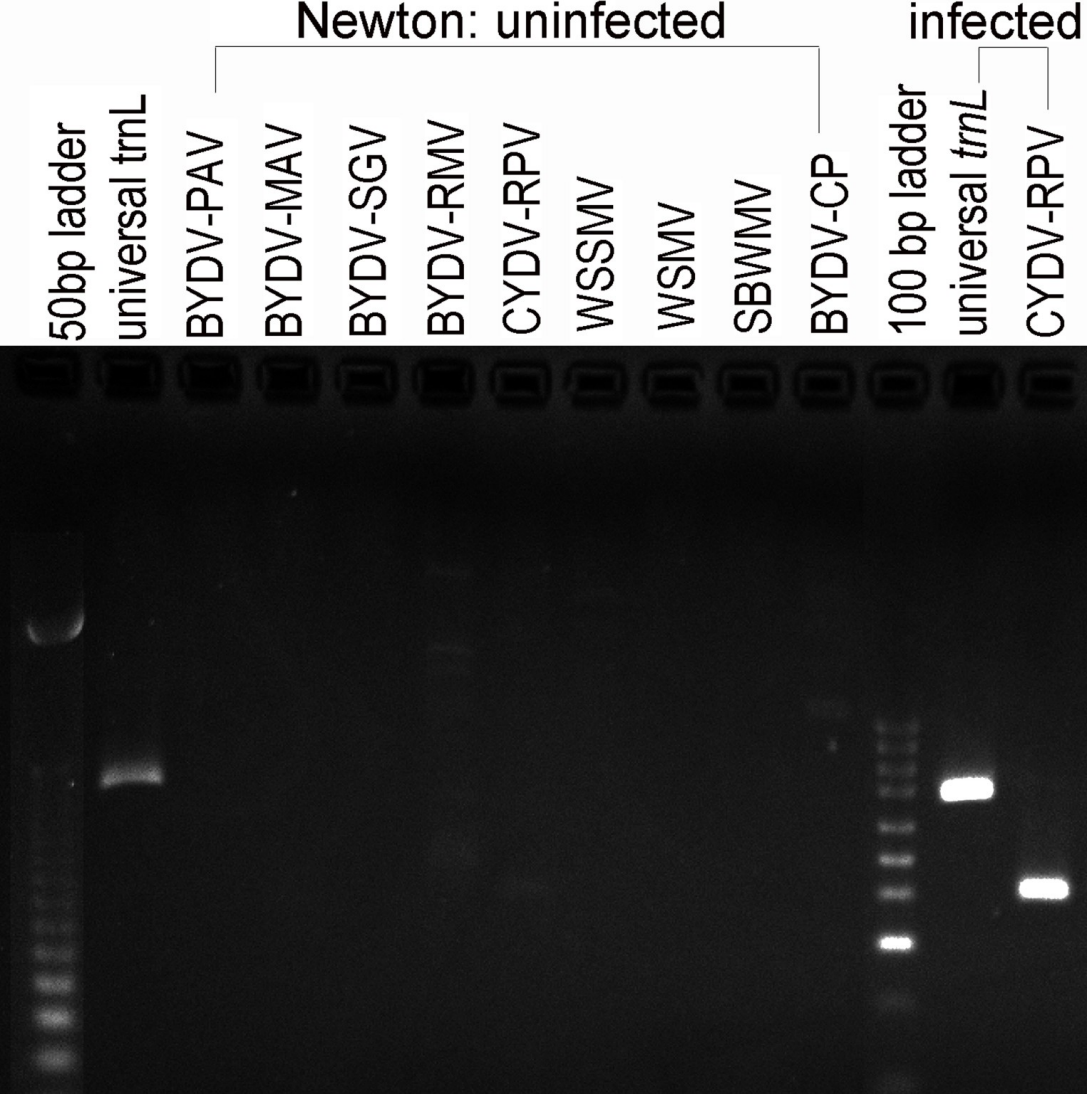
Detection of CYDV-RPV by PCR in infected wheat. The universal trnL primers detected wheat chloroplast DNA. The other primers detected viruses that were absent in uninfected wheat.

**Fig 2:**
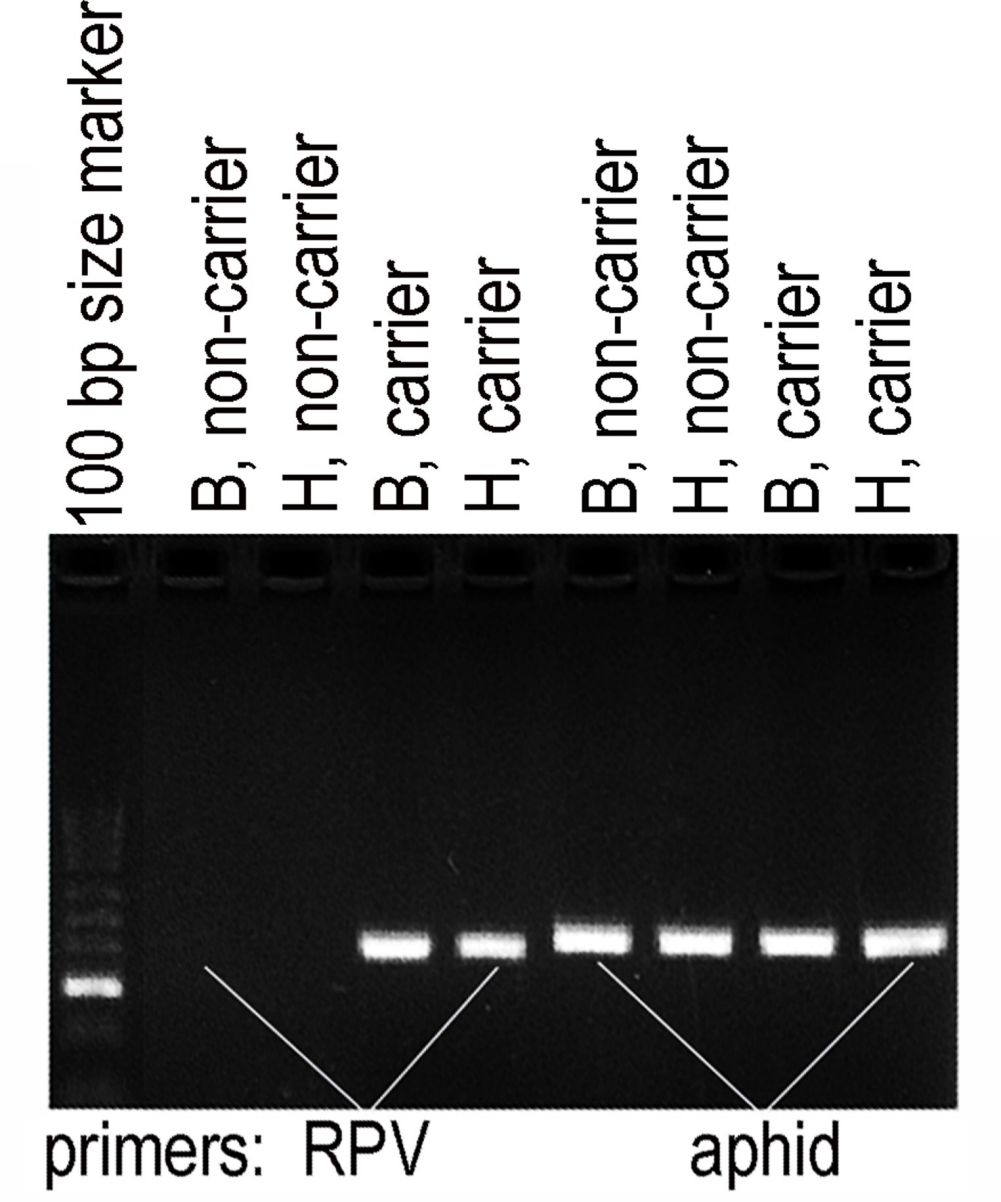
Demonstration that carrier aphids had CYDV-RPV, and non-carrier aphids did not. All amplifications succeeded with aphid-specific primers. Letters B and H designate the two biotypes.

Greenbug biotypes B and H were obtained from Dr. Gary Puterka of the USDA-ARS Wheat, Peanut and Other Field Crops Research Unit in Stillwater, Oklahoma. A single apterous individual of biotype B founded the population of biotype B, and a single apterous individual of biotype H founded the population of biotype H. The parthenogenetic populations built up to several hundred individuals for four to five generations on winter wheat cv. ‘Newton’ in growth chambers at 22°C with a 16 hr light: 8 hr dark photoperiod by cool-white fluorescent light. Without vernalization, Newton did not flower or undergo visible phase change during the experiment. Older wheat plants were removed during maintenance of this population to prevent overpopulation and the production of alate individuals, which itself affects gene expression [12]. Both the founding greenbugs and Newton were free of CYDV (Figs 1 and 2).

### Virus

**Fig 3:**
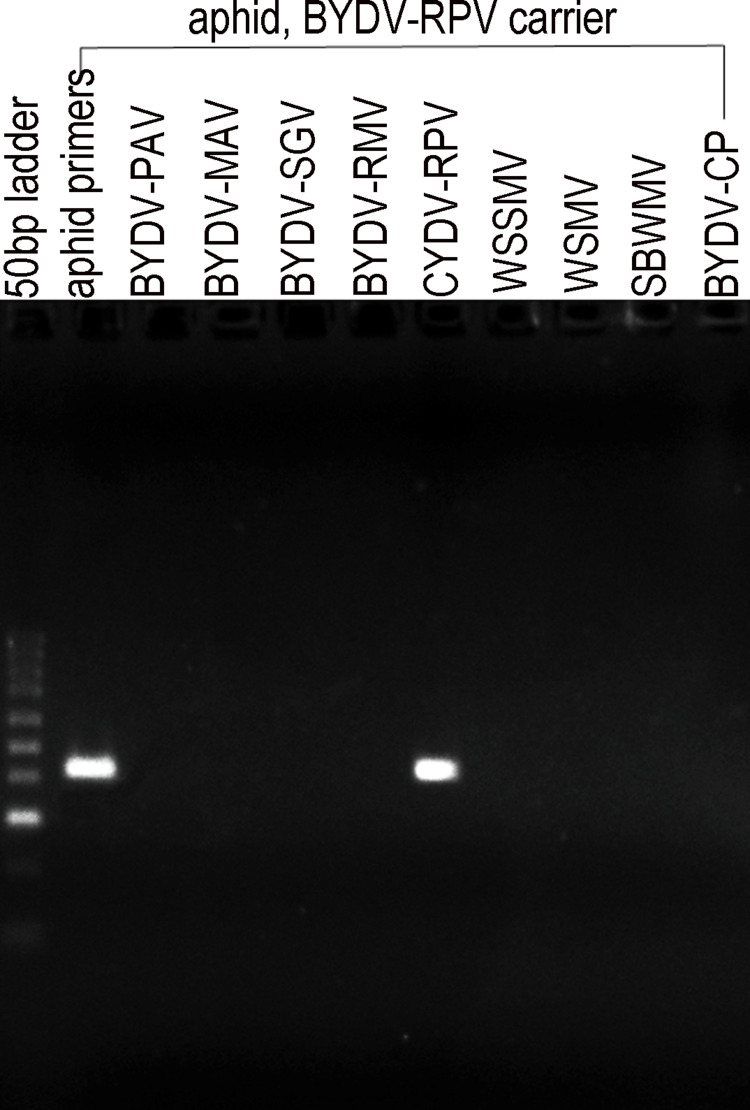
Specific detection of CYDV-RPV by PCR in carrier aphids. Eight other wheat viruses were absent.

About 60 unidentified aphids carrying CYDV-RPV on infected ‘Clintland 120’ oats were obtained from Dr. Steve Scofield (USDA-ARS, West Lafayette, Indiana). These RPV carrier aphids (Fig 3) were introduced to 5–6 day-old wheat cv. ‘Newton’ plants in a separate growth chamber under the same temperature and lighting conditions as above. After a 5-day inoculation access period (IAP) to ensure infection/transmission of RPV to the wheat seedlings and confirm infection by PCR amplification with RPV-specific primers (S1 Table), these aphids and their progeny were manually removed from the wheat plants. The plants were subsequently isolated from all aphids for 23 days with daily monitoring and removal of any missed aphids or their nymphs, until no aphids remained. During this time, the CYDV-RPV infection built up in the wheat plants and visible CYD symptoms developed.

### Infecting greenbugs

Uninfected individuals of greenbug biotype B were placed on two pots of infected ‘Newton’ wheat in one growth chamber, while uninfected individuals of biotype H were placed on two other pots of infected ‘Newton’ in a separate growth chamber. The greenbugs remained there for a 13-day virus acquisition access period (AAP) to acquire RPV from the infected plants (Fig 2) and to increase the population to about 600 individuals per biotype.

### PCR detection of RPV in wheat and greenbug

S1 Table lists nine virus-specific and three universal primer pairs that were used to confirm presence or absence of virus in both wheat and greenbugs at each step before and after infestation. The primers for BYDV-PAV, BYDV-MAV, BYDV-SGV, BYDV-RMV, CYDV-RPV, WSSMV, WSMV, and SBWMV, came from Deb and Anderson [21]; the primers for BYDV-CP came from Jimenez-Martinez et al. [22]. Two positive control primer pairs amplified the *trnL* intron in the chloroplast genome of wheat and a wide variety of other vascular plants, bryophytes, and green algae [23, 24]. A cytochrome oxidase primer pair that works on almost all biotypes of greenbug was used as the greenbug positive control [25].

Total greenbug RNA was extracted using the Qiagen RNeasy Mini Kit (Germantown, MD) following the manufacturer’s instructions. The cDNA was synthesized using the BioRad iScript Advanced cDNA Synthesis Kit (Hercules, CA). The PCR used the MyFi^TM^ DNA polymerase protocol from Bioline (Taunton, MA). The PCR program was: initial denaturation at 95° for 10 min followed by 6 cycles of 95°C for 30s, 60°C for 30s with the annealing temperature decreasing by 1°C in each successive cycle and extension at 72°C for 30s, followed by 30 cycles of 95°C (30s), 55°C (1 min), 72°C (30s) and final elongation at 72°C for 10 min. [21].

### Timed infestation and sample collection

There were three simultaneous replicates for each of eight time points, 0, 1, 2, 3, 5, 10, 15 and 20 days from the beginning. Timepoint 0 was sampled directly from the infected and uninfected maintenance populations described above. At the same time, a leaf segment bearing 20 to 40 apterous greenbugs was placed among the leaves of 12-day-old ‘Newton’ seedlings, which were at the three-leaf stage. The deposited greenbugs were either aviruliferous (control) or viruliferous. Each subsequent collection came from a single pot, which was sampled once and then sacrificed, and each treatment-biotype combination (aviruliferous B, viruliferous B, aviruliferous H, and viruliferous H) was held in a separate growth chamber. Thus 84 pots were sampled overall, plus the initial 12 samples at timepoint 0, for a total of 96 RNA samples. Extra pots of contemporaneous, initially uninfected ‘Newton’ were also available in each chamber. All chambers were maintained at 22°C with a 16 hr light: 8 hr dark photoperiod by cool-white fluorescent lighting.

### RNA sequencing

Greenbugs were sampled for RNA extraction by dropping a leaf segment bearing greenbugs into liquid nitrogen. Frozen aphids were immediately removed from the frozen leaf, counted, and moved to extraction buffer. RNA was extracted with the Qiagen RNeasy Mini kit (Germantown, MD). RNA concentration was measured with a ThermoFisher Scientific Nanodrop spectrophotometer (Waltham, MA). RNA was then stored at -80°C. Samples were then sequenced by Novogene USA (Sacramento, CA). Libraries were prepared successfully for 95 of the 96 samples; the second replication failed for aviruliferous biotype B at day 15. Reads were deposited at NCBI Short Read Archive under bioproject PRJNA981508.

### Bioinformatic analysis

#### Read filtering

Reads files from Novogene USA were processed through HTStream (https://github.com/s4hts/HTStream) without the “superdeduper” step, which would have removed duplicate reads if it had run. HTStream removed adapter, ribosomal RNA, and low-quality or too-short reads. Ribosomal RNA removal was based on matches to NCBI Genbank aphid accessions AH003128.2, AB369153.1, AB369137.1, and S50426.1. Program fastq-pair [26] then separated out all singleton forward or reverse reads. Paired reads files were processed with BBMap reformat.sh [27] to remove empty reads that were rarely left by HTStream and fastq-pair.

The cleaned reads were aligned to two different transcriptomes, designated “KSU” and “BH”. The KSU assembly, which was downloaded from NCBI under master record GIML00000000.1, was a Trinity assembly of 23527 contigs derived from whole-body RNA of *Schizaphis graminum* biotype I at Kansas State University. The BH assembly was a Trinity assembly of 294464 contigs derived from reads of biotype B in nine of the samples in the current study. The samples covered the complete time course and came from both viruliferous and non-viruliferous aphids. Both assemblies were compared with busco [28] to their database hemiptera_odb10. The KSU and BH alignments were processed separately. Because both transcriptomes were derived from whole-body RNA, both contained microbial contigs from the gut, surficial, and endosymbiotic microflora. Both transcriptomes were also aligned with BWA-SW [29] to a panel of 56 databases that represented whole genomes of 35980 bacterial, 531 archaeal, 435 fungal, 10616 viral, and 94 protozoan taxa. Removal of the thus identified contaminant contigs left 23430 contigs in the KSU branch and 258209 contigs in the BH branch. The remaining BH contigs were aligned with the most complete of the available reference genomes at NCBI, GCA_020882235.1, leaving 177199 contigs in the final BH collection.

#### Read alignment and counting

Reads were aligned to the KSU transcriptome with STAR v. 2.7.10a [30] and to the BH transcriptome with bwa mem in bwa v. 0.7.17 [31]. With both transcriptomes, aligned reads were counted with function feature Counts in R package Rsubread version 1.28.1 [32]. Contigs that summed to at least 100 counts over all combinations of timepoint and replicate with either transcriptome were used to produce count matrices for each combination of biotype, viral presence, timepoint, and reference transcriptome. Because of the filtering by count sum, the KSU-B matrix had 11983 hit contigs, the KSU-H matrix had 12525, the BH-B matrix had 81649, and the BH-H matrix had 81485.

#### Principal components analysis

Principal components were found using function princomp in R packages FactoMineR [33] and factoextra [34]. There were two levels of combining counts for each contig: by taking means across all replicates, and by further combining counts across timepoints. The latter used four conditions, biotype B with and without CYDV, and biotype H with and without CYDV. The former used all 32 combinations of two biotypes, two viral statuses, and eight timepoints. The analysis was conducted separately for the KSU and BH transcriptomes.

#### Differential expression

Differential expression was estimated with R package DESeq2, version 1.18.1 [35] with three goals. First, various design formulas were used with the likelihood ratio test to investigate the relative importance of biotype, time, and virus in controlling differential expression. The likelihood ratio test compares a full and reduced model that lacks one or more variables or their interactions. In this case, the count of significantly differentially expressed contigs reflects the importance of the missing variable or interaction; having more differentially expressed contigs implies greater importance when comparing the results with two different reduced models. Second, the count matrices were split by timepoint to identify when genes were most significantly differentially expressed; DESeq2 compared virus to no virus with the Wald test at each individual timepoint. A Perl script was written to identify contigs with a minimum adjusted p-value less than 0.05, and count the instances among them of maximum absolute value of log_2_ fold-change at each timepoint associated with adjusted p-value less than 0.05 in that biotype. Third, DESeq2 compared virus to no virus to group contigs by expression pattern. For each contig with Wald-test adjusted p-value less than 0.05, the expression pattern was a list of log_2_ fold-changes ordered by timepoint. Because there was a gap in maximum abs(log_2_(fold-change)) between 13 and 18, the relatively few contigs above 18 were considered separately as “extrema”. The expression patterns that peaked below 13 were placed in an array for distance calculation with R function dist, hierarchical clustering with R function hclust, separation into 20 clusters with R function cutree, and graphing with R function ggparcoord. The choice of 20 clusters was a compromise between intra-cluster heterogeneity and manageability.

#### Functional analysis

The KSU transcriptome came with functional annotation. Differentially expressed BH contigs were functionally annotated by blastx [36] searches against the NCBI nr protein database at an e-value of 1e-15, using wherever possible the closest hit that did not contain the forbidden strings “ncharacterized”, “ypothetical”, “nknown”, or “nnamed”. Unhit contigs with adjusted p-value less than 0.0001 were searched again against NCBI nr and nt at an e-value of 0.0001 (the identity with p-value was coincidental). Contigs were removed if the second blast’s closest hit did not match an aphid genus. Contigs remained unannotated if there were no hits in nr or if all hits matched the forbidden strings. The taxonomic identity of the closest hit was used to eliminate remaining contaminating BH contigs (mostly wheat and bacteria) from graphing. The filtered KSU contigs were assumed to belong solely to *Schizaphis*. The extrema noted above were also annotated by blastx searches against NCBI nr, and a Perl script found contigs where at least 95% of expression was limited to a single timepoint. Such extrema were counted for each sample to reveal potentially contaminated samples.

The putative effector sequences identified in the first column of Table 1 were downloaded from NCBI and mapped with blastn or tblastn to contigs in the BH transcriptome. These contigs were then followed in DESeq2 output to assess their expression.

**Table 1:** Fold-change and significance of putative effector expression in biotypes B and H.

Accession	Contig name	Log2fc in B	Adj pval in B	Log2fc in H	Adj pval in H
JX134487.1	DN3066_c2_g1_i6	0.606	0.4784	1.834	1.748e-04
JX134489.1	DN32278_c1_g1_i7	-1.045	0.8319	0.743	0.7153
JX134490.1	DN8571_c0_g1_i8	-1.044	0.8357	1.189	0.1061
JX134494.1	DN12630_c0_g1_i8	1.422	0.3072	1.109	0.1764
NC042495.1	DN40164_c0_g1_i2	-1.893	NA	0.734	1.000
NP_001313555.1^a^	DN1178_c0_g1_i1	-1.375	0.6002	4.800	9.097e-05
OK585093.1	DN3944_c0_g1_i2				
ON783485.1	DN122782_c0_g1_i4	-2.247	0.9998	4.675	NA

Effectors were chosen from Atamian et al. [37], Elzinga and Jander [38], Xu et al. [39], Li et al. [40], and Zhang et al. [41, 42]. Shown are log_2_ fold-change and p-value corrected for false discovery rate. There were no hits for accessions DW011417.1, EC389283.1, EE264598.1, JX134488.1, JX134491.1, JX134492.1, JX134493.1, KY986873.1, and XP_015374910.1.

^a^Nine additional accessions mapped most closely to isoforms of DN1178_c0_g1: UXL78742.1, KAE_9543457.1, KAF_0762417.1, VVC_37537.1, XP_022166918.1, XP_025198376.1, XP_025418293.1, XP_026816888.1, and XP_027837168.1. ON783485.1 hit a 60-base motif that is identical in contigs DN1178_c0_g1_i1 and DN122782_c0_g1_i4. No isoform of contig DN3944_c0_g1 had enough counts to be processed with DESeq2.

## Results

### Overview

In total, 1,840,820,000 raw reads, yielding 1,000,090,000 clean reads, were obtained from 95 samples (S2 Table). The ribosomal 18S-28S RNA (rRNA) frequency ranged from 15% to 88.5% among samples, with a mean of 42.4%. Zhao et al. [43] reported that rRNA comprises 80–90% of the RNA in animal cells. Thus the library preparation protocol partially selected against rRNA. Library construction failed for one sample, B62, representing the second replicate for aviruliferous biotype B at day 15. Limited starting material (only two aphids) was available for another sample, HRPV43, which represented the third replicate of viruliferous biotype H at day 3.

The BH Trinity assembly consisted of 294464 contigs. Its mean contig length was 786.8, median was 349, and N50 was 1616. There were 294464 isoforms in 209467 genes and thus 1.41 isoforms per gene. It covered 99.0% of the highly conserved genes in busco database hemiptera_odb10, but 88.1% were duplicated in coverage, 10.9% were single, and 0.4% were fragmented. In contrast, the KSU assembly consisted of 23527 contigs. Its mean contig length was 2171.7, median was 1799, and N50 was 2823. There were 23527 isoforms for 13263 genes, thus 1.77 isoforms per gene. It covered 96.7% of the genes in busco database hemiptera_odb10; 47.5% were duplicated in coverage, 49.2% were single, and 0.9% were fragmented. Removal of microbial contigs from the BH assembly left 258209 contigs, and 169686 of these contigs had at least 100 read counts over all timepoints. Alternatively, alignment of all 294464 contigs to the *Schizaphis graminum* reference genome identified 177199 matching contigs at 1e-15, including matches as short as 55 bases. Among these, 72786 contigs had at least a sum of 100 reads over all timepoints in biotype B, and 74262 had at least 100 in biotype H. There were 68288 contigs with at least 100 in each biotype, 4498 with this many only in biotype B, and 5974 with this many only in biotype H.

Total mapping was estimated by summing DESeq2 base expression for each assembly over all combinations of biotype, time, and viral carrier status. The totals were 276902591 for the KSU assembly and 774990387 for the BH assembly. Retaining only contigs that mapped to aphid genes reduced the total BH count to 137273892. These totals applied for the same read set over both transcriptomes.

**Fig 4:**
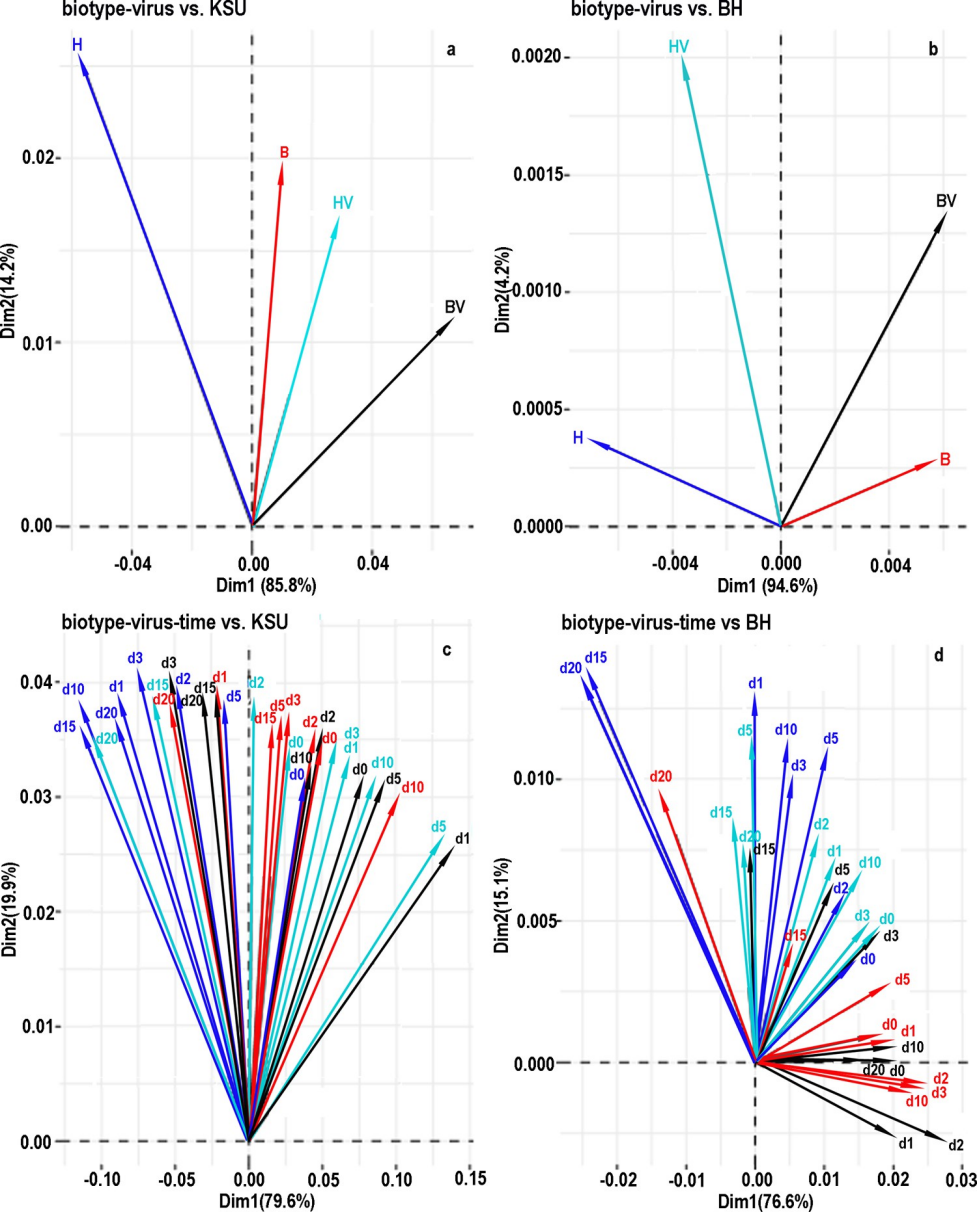
Results of principal components analysis for combinations of biotype and virus and of biotype, virus, and time. Results are plotted against the first and second principal components for the KSU transcriptome at left and the BH transcriptome at right. Each color represents a combination of biotype and carrier status. The color scheme was constant over all four panels of the figure.

Principal components gave an overview of the effects of biotype and virus on gene expression. Four analyses were performed, separate and merged across timepoints for biotypes B and H. For time-merged counts against the KSU transcriptome (Fig 4A), the first principal component separated virus-free from viruliferous aphids and accounted for 86% of the variance. This indicated that virus influenced gene expression more than biotype. In contrast, with the BH transcriptome (Fig 4B), the first principal component mostly separated biotypes and accounted for 95% of the variance, and the second principal component mostly separated viruliferous from virus-free. When timepoints were separated (Fig 4C), biotype H tended to occupy the lower range of the first principal component while biotype B occupied the upper range. This tendency was greater with the BH transcriptome (Fig 4D). There was no obvious segregation by timepoint along either principal component with either transcriptome.

**Fig 5:**
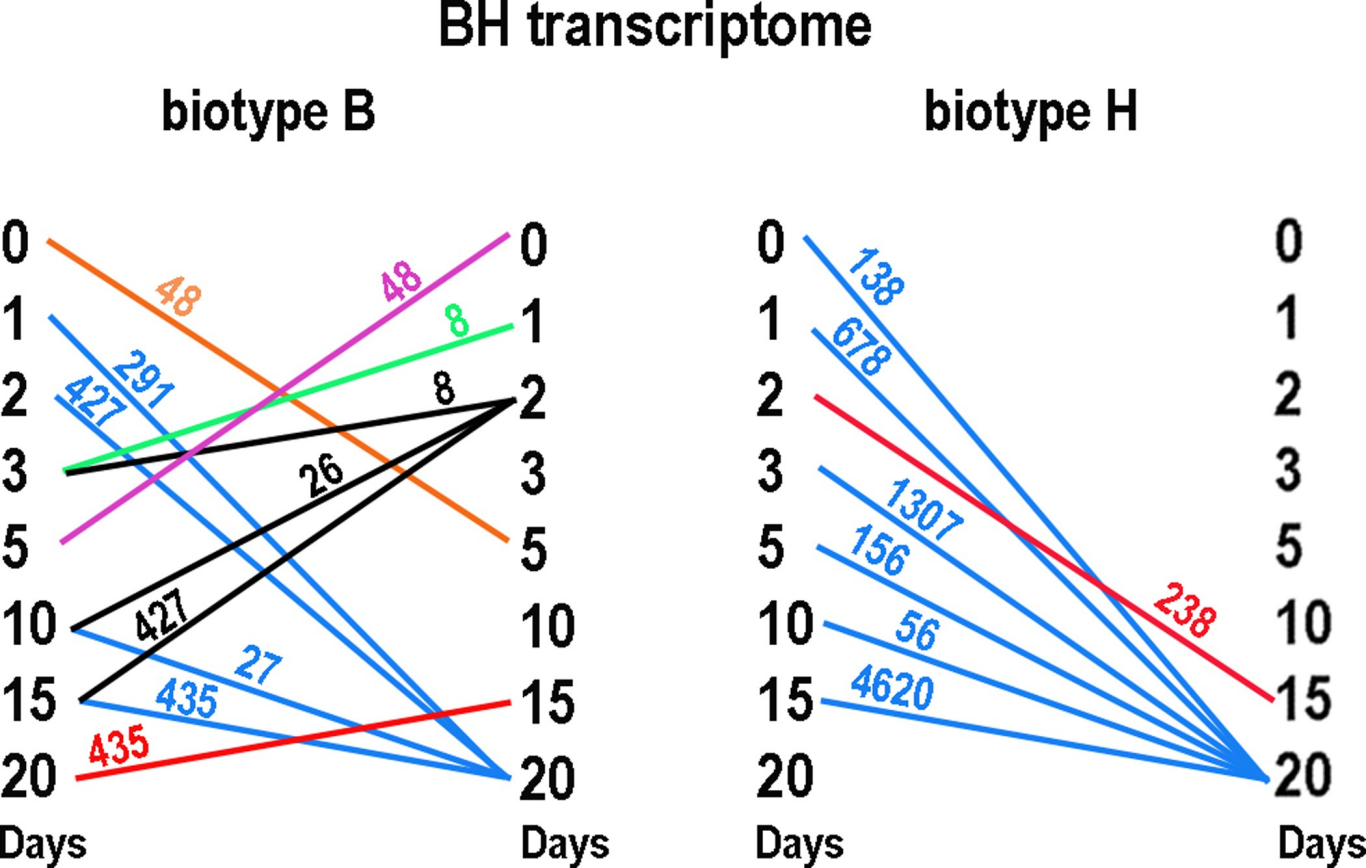
Pairs of most similar dates when viruliferous and aviruliferous greenbugs were compared across timepoints. Each count is the number of contigs that were differentially expressed at both timepoints. The timepoint in the left column shared the indicated count with the timepoint in the right column. The colors merely relate a count to the timepoints compared.

Another way to assess similarity among samples is to count the contigs that are differentially expressed for virus versus no virus in both samples. These counts are displayed in Fig 5 by date versus the BH transcriptome. In Fig 5, dates in the left column are most similar to dates in the right column as connected by line segments. The closest relationships were not necessarily reciprocal, e.g., day 3 with biotype B was most similar to days 1 and 2, but those dates themselves were most similar to day 20. The relationship was simpler for biotype H, where all other days but day 2 were most similar to day 20, possibly because so many contigs were differentially expressed on day 20.

**Fig 6:**
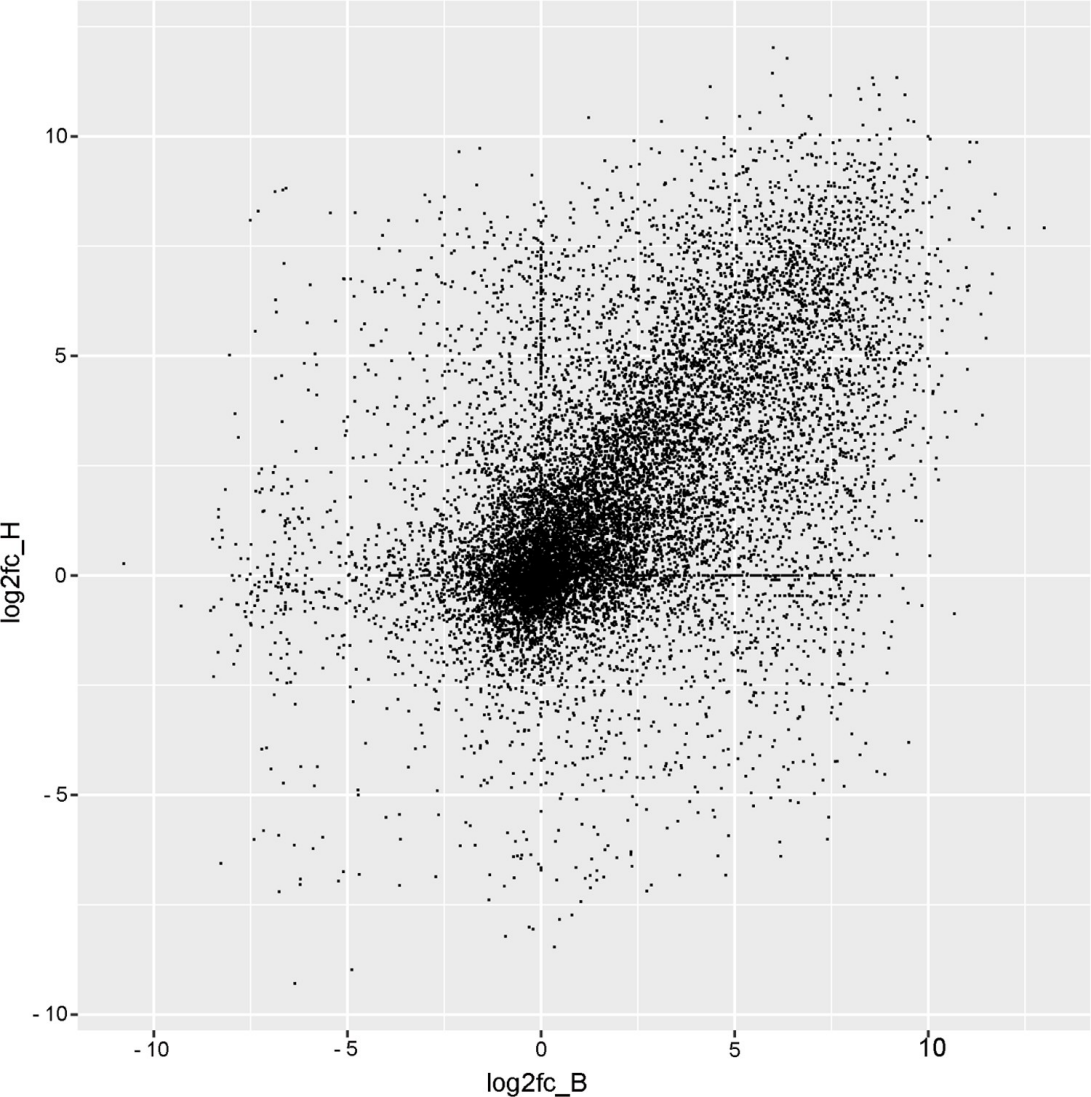
Log_2_ fold-change in biotype H versus log_2_ fold-change in biotype B for BH contigs differentially expressed at p < 0.05 in at least one time point.

Another reflection of the different responses of biotypes B and H is to plot log_2_ fold-change for biotype H against log_2_ fold-change for biotype B for the same contig at the same timepoint (Fig 6). While points are scattered in all four quadrants of the figure, denoting many contigs with up-regulation in one biotype and down-regulation in the other biotype, there is a majority of contigs that plotted near the diagonal in the upper right quadrant. Pearson’s correlation coefficient for the points in Fig 6 was 0.500 (p = 2.2e-16, df = 14606), which indicates a moderate correlation of response in biotype H to response in biotype B. The denser rows of points along the upper y-axis and right-hand x-axis represent instances where one of the fold-changes was NA, mathematically undefined.

### Results of likelihood ratio tests

The relative importance of biotype, time, and viral presence was investigated with the 15 likelihood ratio tests listed in Table 2. The table contains columns of complete and reduced design formulas in the format “~ variable + another variable”, and interactions are formatted as “variable1:variable2”. A “~ 1” indicates uniform expression across all timepoints or biotypes or viral presence. Counts of significantly differentially expressed contigs are given as is and as fractions of total count of contigs to account for variation in total counts among treatments. These tests were applied to all the data put together and separated by only one or two factors; thus a comparison of virus to uniform expression took counts with virus over both biotypes and all timepoints, and compared them to counts without virus over both biotypes and all timepoints.

**Table 2:** Results of likelihood ratio tests.

Full model	Reduced model	KSU transcriptome		BH transcriptome	
		Count of significant contigs	Fraction of significant contigs	Count of significant contigs	Fraction of significant contigs
~ biotype	~ 1	2057	0.1331	20867	0.1233
~ time	~ 1	2288	0.1481	17289	0.1021
~ virus	~ 1	292	0.0189	20289	0.1199
~ biotype + virus	~ 1	2191	0.1418	40094	0.2369
~ time + biotype	~ 1	3764	0.2436	35119	0.2075
~ time + virus	~ 1	2573	0.1665	29372	0.1735
~ biotype + virus	~ biotype	305	0.0197	20209	0.1194
~ biotype + virus	~ virus	2095	0.1356	20962	0.1238
~ time + biotype	~ biotype	2401	0.1554	19360	0.1144
~ time + biotype	~ time	2161	0.1398	20912	0.1235
~ time + virus	~ time	312	0.0202	14242	0.0841
~ time + virus	~ virus	2265	0.1466	16757	0.0990
~ biotype + virus + biotype:virus	~ biotype + virus	27	0.0017	83	0.0005
~ time + biotype + time:biotype	~ time + biotype	24	0.0016	325	0.0019
~ time + virus + time:virus	~ time + virus	38	0.0025	705	0.0042

The reduced model “~ 1” represents constant expression.

The results differed between the two transcriptomes, which differed in contig count, coverage, and identity with the biotype B and H genomes. The top three rows demonstrate that virus was less important than time or biotype with the KSU transcriptome, while all three factors were about equally important with the BH transcriptome, i.e., the same read set gave different results with the two transcriptomes. In the next three rows, combining virus with either biotype or time did not change the response much with the KSU transcriptome, whereas biotype plus time almost doubled the response of either alone. In contrast, all three factor pairs increased the response versus any single factor with the BH transcriptome. The implication is that mostly distinct sets of contigs were responding to time and biotype with KSU, or responding to any factor pair with BH.

In rows 7 through 12, pairs of factors were compared with a single factor. Omitting virus greatly reduced the count of significant contigs with the KSU transcriptome but did not reduce the count as much with the BH transcriptome. Instead, each factor’s contig set was relatively independent of the other two with BH. Finally, in the last three rows, the interactions between any two factors were unimportant with either transcriptome. This of course does not preclude major effects from single genes that respond to any of the factors.

### Timing of most deviant expression

For each contig there existed a timepoint where abs(log_2_(fold-change)) was maximal. With the KSU transcriptome, there were 3187 contigs for which the Wald-adjusted p-value was less than 0.05 for at least one time point in each biotype, although this was not necessarily the same timepoint in both biotypes. Table 3 gives the distribution of the dates of maximal abs(log_2_(fold-change)) for biotypes B and H. It also provides summed counts of peaking contigs for each date and each biotype.

**Table 3:** Counts of contigs whose expression differential peaked on each date in each biotype versus the KSU transcriptome.

Days	H-0	H-1	H-2	H-3	H-5	H-10	H-15	H-20	Row sum
B-0	0	1	2	10	6	1	2	10	32
B-1	16	14	13	20	17	8	59	127	274
B-2	8	18	8	12	5	10	80	248	389
B-3	1	0	2	10	2	0	7	7	29
B-5	3	4	9	6	47	3	11	123	206
B-10	1	0	4	1	15	2	6	12	41
B-15	7	41	6	24	5	3	220	591	897
B-20	18	15	17	72	41	4	329	823	1319
Column sum	54	93	61	155	138	31	714	1941	

Rows represent biotype B and columns represent biotype H. For example, there were 59 contigs with most differential expression on day 1 in biotype B and day 15 in biotype H.

The distribution is skewed heavily to 15 and 20 days after initial infestation, with 70 percent of peak dates for biotype B and 83 percent for biotype H at these two times. There are minima for biotype B on day 3, biotype H on day 2, and both biotypes on day 10. Day 0 has few peak dates. The high percentage of peak dates at days 15 and 20 accompanied severe yellow dwarf symptoms in the infected host plants and senescence under aphid feeding in both infected and uninfected host plants. The increased count of peak dates on days 1 to 3 did not accompany visible changes in the host. S3 and S4 Tables list annotated contigs that peaked at each timepoint. The listing is ordered by minimum Wald-adjusted p-value.

### Clustering of expression patterns

**Fig 7:**
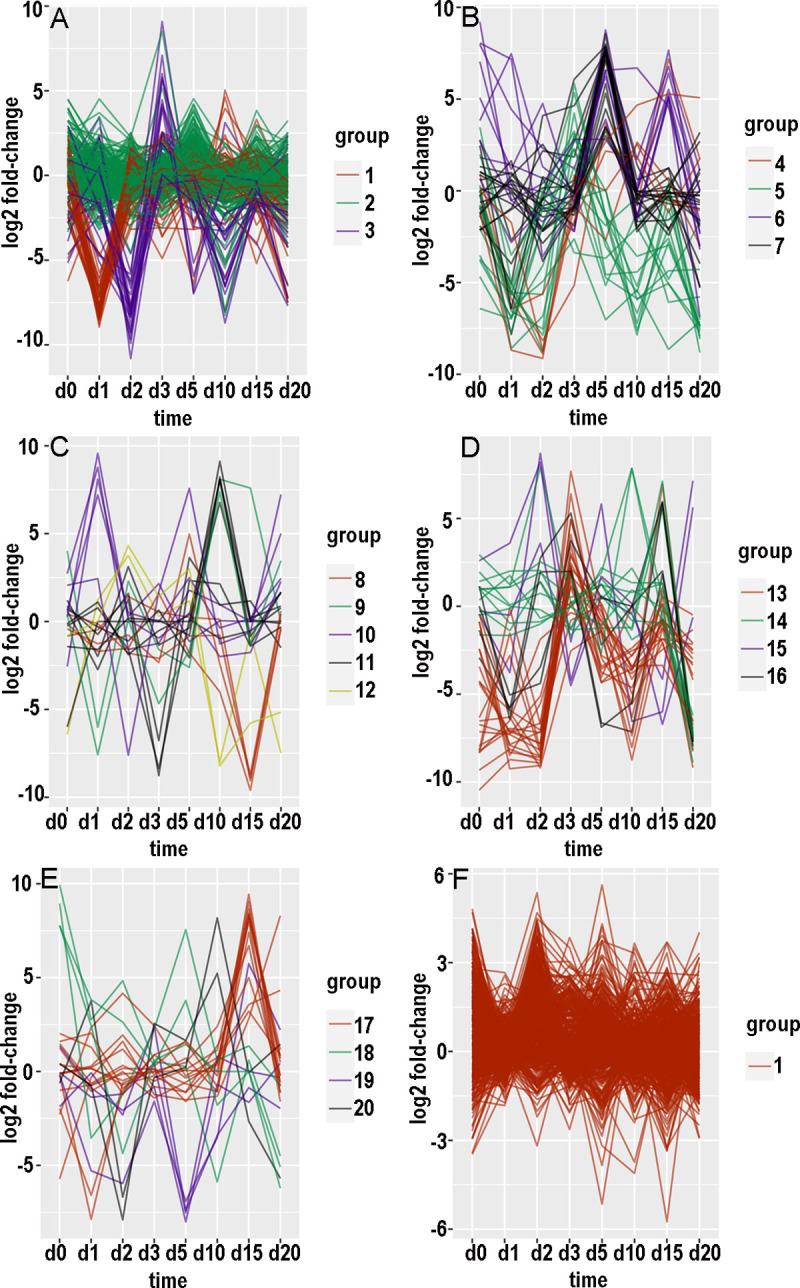
Graphical displays of clustered expression profiles. Log_2_ fold-change is plotted against day of sampling.

**Fig 8:**
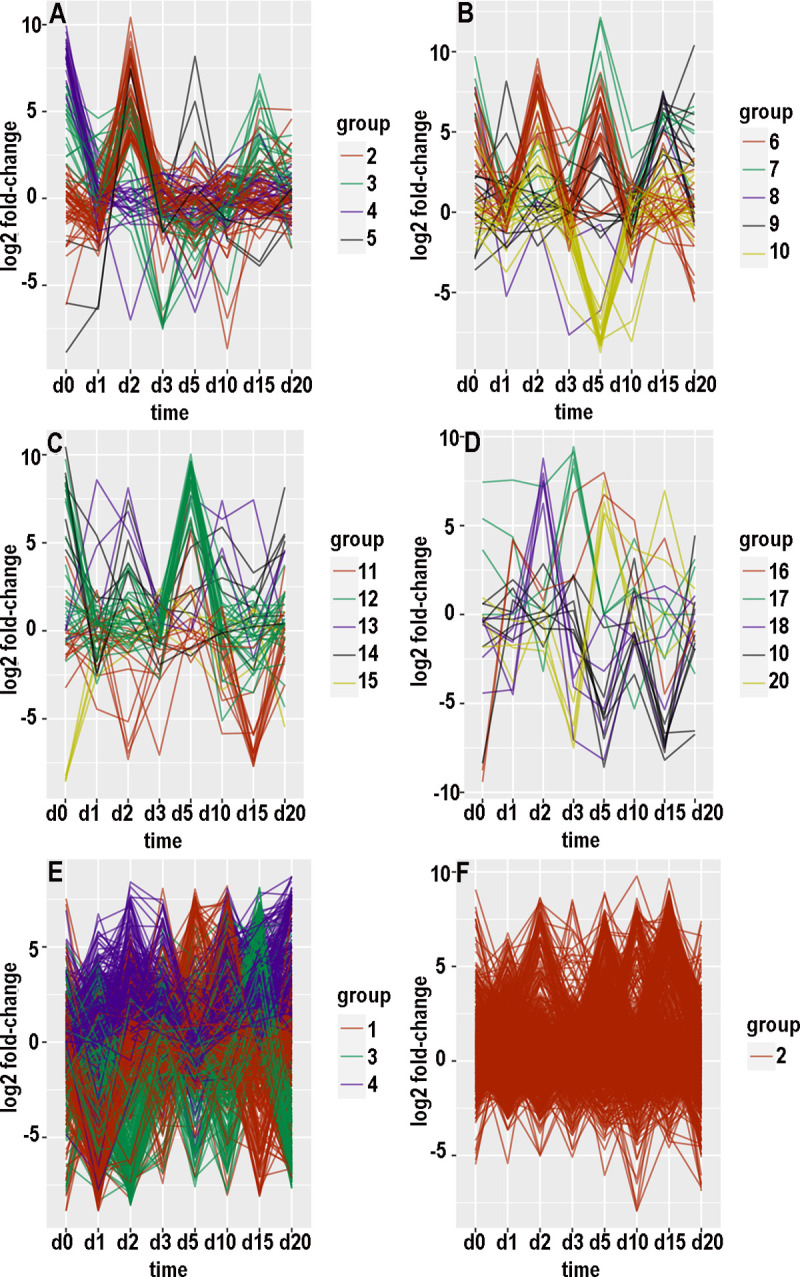
Graphical displays of clustered expression profiles. Log_2_ fold-change is plotted against day of sampling.

**Fig 9:**
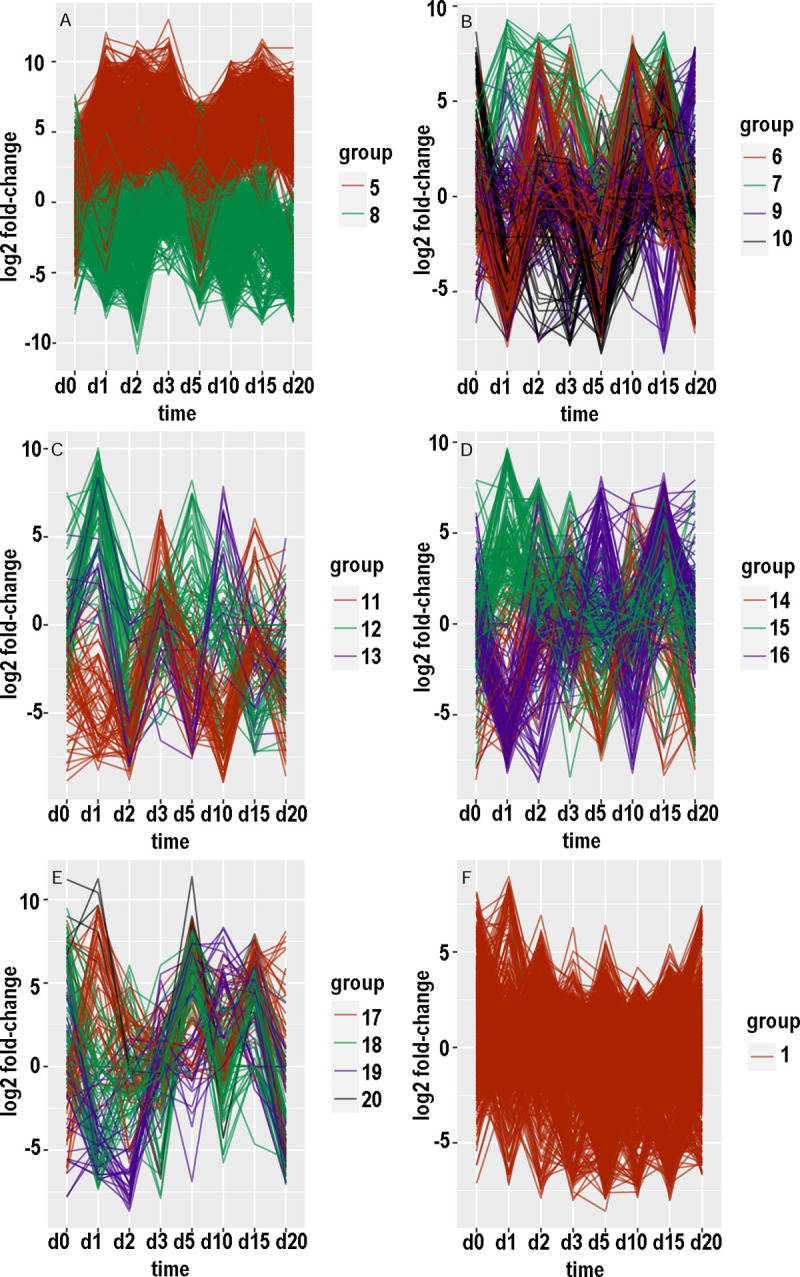
Graphical displays of clustered expression profiles. Log_2_ fold-change is plotted against day of sampling.

**Fig 10:**
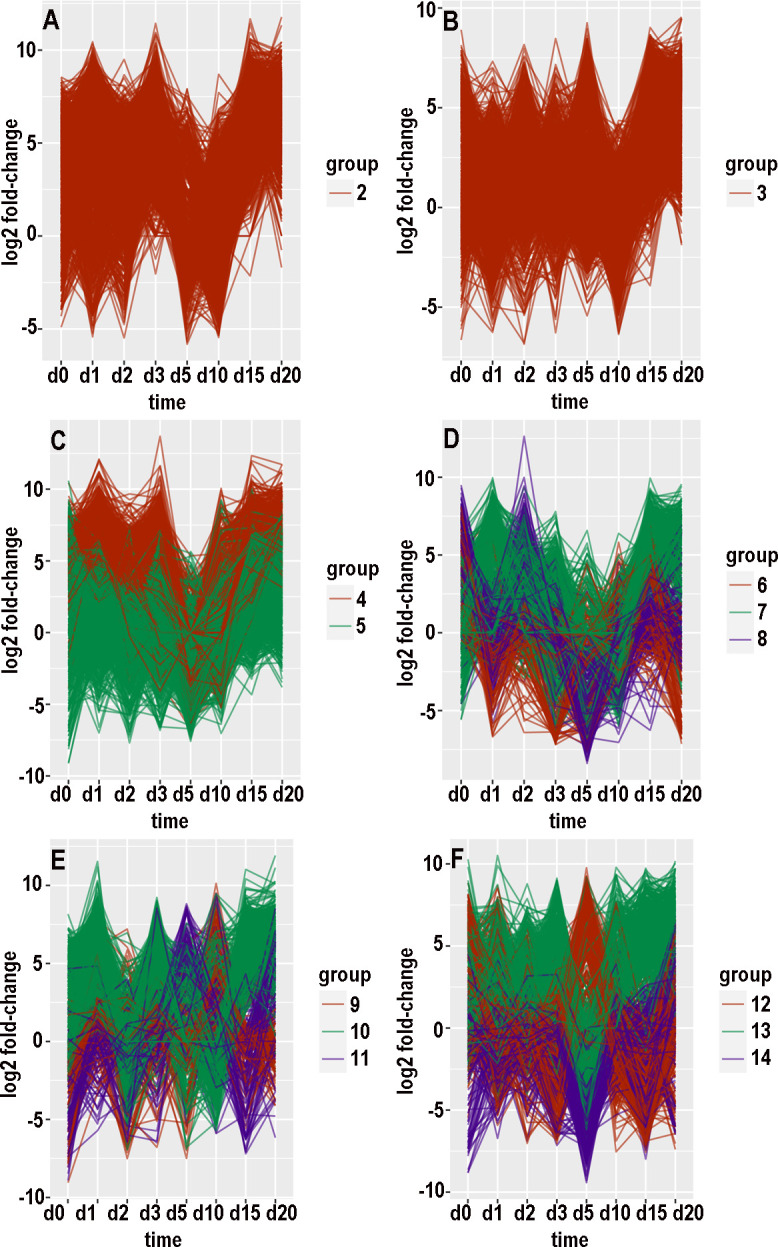
Graphical displays of clustered expression profiles. Log_2_ fold-change is plotted against day of sampling.

**Fig 11:**
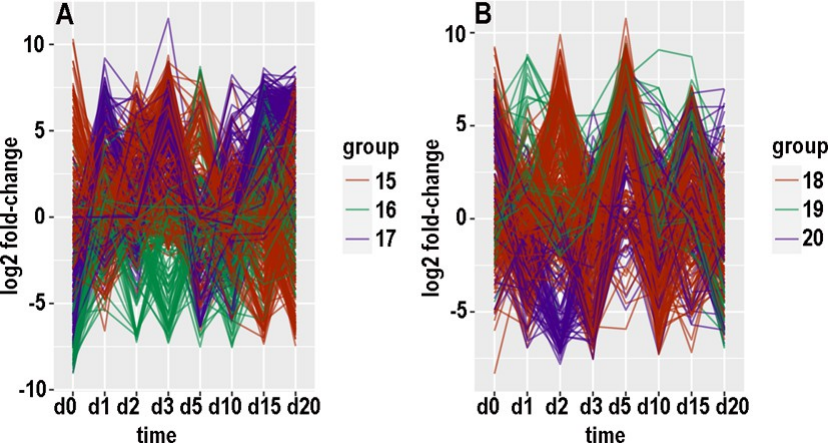
Graphical displays of clustered expression profiles. Log_2_ fold-change is plotted against day of sampling.

There were 15454 contigs with at least 100 counts over the time course in the KSU transcriptome and 169264 such contigs in the BH transcriptome. For each of these contigs, an expression pattern was defined as a list of the value of log_2_(fold-change) at consecutive timepoints. Hierarchical clustering and tree-cutting yielded groups of similarly expressed contigs that had been selected for maximum abs(log_2_(fold-change)) less than 14. Grouping was arbitrarily limited to 20 groups even though some groups still had many hundreds of contigs and remained visibly heterogeneous when plotted as log_2_(fold-change) versus timepoint. Plotting log_2_ fold-changes for the biotypes and transcriptomes separately with small numbers of groups at a time resulted in 26 graphs (panels A through F in Figs 7–10, panels A and B in Fig 11). S5–S8 Tables provide annotations and minimal adjusted p-values of the contigs in each group. S9 through S12 Tables summarize the number of contigs, timepoint of the most maxima, and timepoint of the most minima, in each group. In the following paragraphs that describe the groups, “up-regulated” and “increased” refer to increased expression with CYDV, “minimal” refers to decreased expression with CYDV, and top ranking refers to minimal adjusted p-value. “Peaks” and “dips” refer respectively to log_2_ fold-changes greater than 3 or less than -3. “Low expression” refers to base expression less than about 150 counts, “high expression” refers to base expression greater than about 600 counts, and “intermediate expression” is in between.

#### Biotype B, transcriptome KSU

Group 1 (Fig 7A) was the second most populated group and had reduced expression with virus on day 1 and less so on day 20. Expression was enhanced somewhat on day 10. Among the top 10 genes for minimal adjusted p-value, one was unannotated and three were hypothetical proteins of unknown function. The functions of the other six were seemingly unrelated to one another, but one was a stress protein and two were apparent transcription factors. Group 2 (Fig 7A) was the most populated group and had relatively high base expression and even expression across the time course with minor maxima on days 5, 0, 15, and 20. Four of the contigs with least p-value were hypothetical proteins. Actin, a PDZ-LIM-domain protein, trehalose transporter Tret1, acylphosphatase-2, and an acyl-CoA dehydrogenase were all up-regulated, while a peroxidase was down-regulated. Group 3 (Fig 7A) was strongly down-regulated on day 2 and upregulated on day 3. Six of the top ten contigs coded hypothetical proteins, and one was unannotated. The other three were related to retroviruses or retrotransposons. All but one had relatively low base expression. Group 4 (Fig 7B) consisted of five contigs that were strongly down-regulated on days 2 and 3. Only two had a known function, which can be considered housekeeping, and all five had low base expression.

Group 5 (Fig 7B) consisted of 14 contigs that were up-regulated on day 3 and down-regulated on days 1, 2, 10, and 20. Four of the top 10 coded hypothetical proteins, two were involved with transposable elements, and the other four had seemingly unrelated functions. Base expression was low for the whole group. Group 6 (Fig 7B) consisted of nine contigs that were upregulated at days 0, 5, and 15. There were dips to equal expression with and without virus on days 10 and 20. One contig was unannotated, two encoded hypothetical proteins, two encoded protein kinases, and the other four had diverse functions. Most notable was flightin, a protein specific to insect flight muscle that indicated a transition to the alate form, which peaked at day 10. Base expression was high for flightin and two of the hypothetical proteins and low for the other contigs. Group 7 (Fig 7B) consisted of 19 contigs that were strongly upregulated on day 5. There were six hypothetical proteins among the top 10 contigs. Two other contigs encoded sclerostin, a protein that otherwise inhibits bone formation in vertebrates. The other two contigs encoded a thiamine transporter and a piggy-bac transposon protein. Expression was high only for three of the hypothetical proteins.

Group 8 (Fig 7C) consisted of a serine protease and three hypothetical proteins. Their expression was greatly reduced on day 15. Expression was moderate in one hypothetical protein and low for the rest. Group 9 (Fig 7C) consisted of one unannotated and one hypothetical protein whose weak expression was minimal on day 1 and maximal on day 10. Group 10 (Fig 7C) consisted of three hypothetical proteins, a phosphoesterase, and a polypeptide N-acetylgalactosaminyltransferase. Base expression was weak but up-regulated on days 1 and 20. Group 11 (Fig 7C) consisted of eight weakly expressed contigs that encoded two hypothetical proteins and six other proteins of diverse function. Their base expression was minimal on day 3 and maximal on day 10. Group 12 (Fig 7C) consisted of two functionally unrelated, weakly expressed contigs with maximal expression on day 2 and minimal expression on days 10 and 20. Group 13 (Fig 7D) consisted of 20 weakly expressed contigs with increased expression on day 3 and minimal expression on days 1, 2, 10, and 20. Five of the top 10 differentially expressed contigs encoded hypothetical proteins; two encoded transcription factors, and three apparently belonged to transposable elements.

Group 14 (Fig 7D) consisted of 11 weakly expressed contigs that were most uniformly down-regulated on day 20. Some were up-regulated on day 10 or 15. Four encoded hypothetical proteins. One protein had a VPS9 domain and was potentially involved in signaling cascades. Another protein was a membrane-bound tyrosine kinase receptor that might bind hormones. Two more proteins acted in the nucleus. One protein induced apoptosis, while another possibly synthesized heparin. The eleventh overcame the effects of brefeldin A, which can inhibit the formation of vesicles from endoplasmic reticulum. Group 15 (Fig 7D) consisted of three weakly expressed contigs that were up-regulated on day 2 and down-regulated on days 3 and 15. One of them, primosomal protein 1, was probably bacterial and involved in DNA replication. Another protein shuttled a translation initiation factor between the nucleus and cytoplasm. The third was a hypothetical protein. Group 16 (Fig 7D) consisted of four weakly expressed contigs whose expression was reduced at days 1, 5, and 20, and increased at days 3 and 15. The proteins were a CGG repeat-binding protein, a fork head domain protein (a possible transcription factor), a non-autonomous transposon protein, and a hypothetical protein.

Group 17 (Fig 7E) consisted of 14 contigs with mostly intermediate base expression that peaked on day 15. Perhaps the most interesting of the group were two genes for vacuolar protein sorting-associated protein and one for TSSC1, a protein involved in endosome retrieval. Others included semaphorin, a metaphase inducer, a subunit of a transcription complex, a “dead ringer” DNA-binding protein, cystathionine gamma-lyase, and a hypothetical protein. Group 18 (Fig 7E) consisted of four weakly expressed, functionally unrelated proteins whose expression peaked at day 0 and was minimal at day 20. Group 19 (Fig 7E) consisted of four weakly expressed proteins whose expression was minimal on day 5. Two of these proteins interacted with RNA, and a third one oxidized formaldehyde to formic acid. Group 20 (Fig 7E) consisted of two weakly expressed proteins whose expression was minimal on day 2 and maximal on day 10.

#### Biotype H, transcriptome KSU

Group 1 (Fig 7F) consisted of 546 mostly highly expressed contigs with relatively even expression. A substantial number of these peaked on days 0 or 2. Among the top 10 were two contigs that encode cathepsin B, and a third cathepsin B ranked thirteenth. The top 10 also included aminopeptidase N, protein yellow, ADP-ATP carrier protein, two hypothetical proteins, and two proteins that elongate long-chain fatty acids. Group 2 (Fig 8A) consisted of 50 mostly intermediately to highly expressed contigs whose expression peaked on day 2 and was mostly lowest on day 1. Eight of the top ten were hypothetical proteins. The other two were sodium-potassium-calcium exchanger 5 and a collagen-derived phosphorylase. Group 3 (Fig 8A) consisted of 20 intermediately to highly expressed contigs that dipped on day 3 and peaked on days 0 and 15. Six of the top ten mapped to cathepsin B. Two of the top 11 were UDP-glucuronosyltransferase. The other three were a lysosomal endopeptidase, a lipase, and a hypothetical protein. Group 4 (Fig 8A) consisted of 23 contigs with low to intermediate expression. Five of the top ten encoded hypothetical proteins. The others were pupal cuticle protein, a lipid phosphatase, a keratin-binding protein, acetyl coenzyme A carboxylase, and a transcription factor. Group 5 (Fig 8A) consisted of three weakly expressed, functionally unrelated contigs with minimal expression on days 0 and 1 and high expression on days 2 and 5.

Group 6 (Fig 8B) consisted of 22 mostly weakly expressed contigs whose peak expression was on days 0, 2, and 5. Expression was mostly least at day 20. Four of the top 11 contigs encoded hypothetical proteins. The other functions were diverse, but one was trehalose transporter Tret1, which also appeared with group 2 for biotype B.

Group 7 (Fig 8B) consisted of five contigs, of which two were highly expressed. Expression dipped on day 1 and peaked on days 0 and 5. Only one had a known function, a facilitator of transport of small molecules across membranes. Group 8 (Fig 8B) consisted of two weakly expressed, functionally unrelated contigs whose expression peaked on day 15. Group 9 (Fig 8B) consisted of 11 weakly to intermediately expressed contigs that peaked on day 15 and dipped on day 10. Four of the proteins bound to nucleic acids, two were involved with vesicles, two were hypothetical proteins, one was involved in signaling, one was a cytochrome P450, and one was a 28S ribosomal protein. Group 10 (Fig 8B) consisted of 17 contigs with mostly intermediate expression that prominently dipped on day 5. Most of them peaked on day 2. Three of the top 11 contigs encoded hypothetical proteins, while two encoded cathepsin B and two others encoded zinc finger proteins. Others included cytohesin 1, a spliceosome protein, glycogen synthase, and a cytochrome P450. Group 11 (Fig 8C) consisted of 14 contigs with low to intermediate expression that prominently dipped on day 15. Some of them also dipped on days 2 or 3. The top ten contigs included two zinc-finger proteins, a 60S ribosomal protein, an exoribonuclease, a heat-shock protein, a tRNA methyltransferase, an enabler of phosphatase binding, and a regulator of autophagy.

Group 12 (Fig 8C) consisted of 27 weakly to intermediately expressed contigs with a prominent peak at day 5. Some also peaked on day 0. Their diverse functions ranged from actin fibril assembly to esterase to regulation of insect tracheal development. Group 13 (Fig 8C) consisted of three weakly expressed, functionally diverse contigs that peaked on days 2 and 10. Group 14 (Fig 8C) consisted of seven mostly highly expressed contigs that peaked on day 0 and dipped on day 1. The only three known functions involved actin filaments, cuticle structure, and the ABC transporter. Group 15 (Fig 8C) consisted of four weakly expressed contigs with minimal expression on day 0. The only suspected function was nicotinate-nucleotide adenylyltransferase. Group 16 (Fig 8D) consisted of two weakly expressed, functionally unrelated contigs that were minimal on day 0 and peaked on days 1 and 5. Group 17 (Fig 8D) consisted of four weakly expressed contigs whose differential expression peaked on day 3 and fell off by day 5. The only known functions were thiamine transport and DNA binding. Group 18 (Fig 8D) consisted of five weakly expressed contigs. Two encoded hypothetical proteins, two others encoded potassium channels, and the fifth protein associated with the cytoskeleton. Group 19 (Fig 8D) consisted of seven functionally diverse, weakly to intermediately expressed contigs whose expression dipped on days 5 and 15. Functions included modulation of ubiquinylation, heat-shock response, signal transduction, RNA splicing and silencing, and facilitating the assembly of multimeric protein complexes. Group 20 (Fig 8D) consisted of four weakly expressed, functionally diverse contigs whose expression dipped on day 3 and peaked on day 5. One contig encoded a cytochrome P450, another helped to organize peroxisomes, and a third mediated transcription by RNA polymerase II.

#### Transcriptome BH

The count of differentially expressed KSU contigs was 394 for biotype B and 778 for biotype H. After removal of known contaminants, the counts for differentially expressed BH contigs were 4631 for biotype B (11.75 times greater) and 16789 for biotype H (21.58 times greater). The more populated clusters were visibly heterogeneous (e.g., Fig 9F) despite being separated from other clusters.

An itemized account of each group was not attempted. Instead, the general shape of each group’s graph is provided to augment S11 and S12 Tables. With biotype B, group 1 (Fig 8E) had a minimum on day 1, and many of the contigs peaked on day 5 or 10. Group 2 (Fig 8F) was relatively even across the timepoints; the most pronounced maximum was on day 15, and some contigs peaked on day 5 or 10. Group 3 (Fig 8E) peaked on day 15 and dipped on day 2, and some contigs dipped on day 5, 10, or 20. Group 4 (Fig 8E) had peaks of subsets of contigs on days 1, 2, 3, and 20, and there were no pronounced dips. Group 5 (Fig 9A) was elevated with virus throughout except day 5, and some contigs were low at day 0. Group 6 (Fig 9B) had peaks on day 2 or 3 and again on 10 or 15 for subsets of contigs, and there were dips on days 1, 5, and 20. Group 7 (Fig 9B) had subsets that peaked on days 1, 2, or 3, and again on days 10 or 15; there was a dip on day 20. Group 8 (Fig 9A) expression was reduced with virus at all timepoints except day 3 and somewhat on day 0. Group 9 (Fig 9B) peaked on day 20 and dipped on day 15. Group 10 (Fig 9B) peaked on days 0 and 15 and dipped on day 1; subsets also dipped on day 3 or 5. Group 11 (Fig 9C) peaked on day 3 and to a lesser extent day 15, while subsets dipped on day 1 or 2, day 10, and day 20. Group 12 (Fig 9C) peaked on days 1 and 5 and dipped on days 2 and 15. Group 13 (Fig 9C) peaked on days 1 and 10 and dipped on days 2 and 5. Group 14 (Fig 9D) dipped on day 5, and subsets peaked on day 2 or 3, peaked on day 10 or 15, dipped on day 0 or 1, and dipped on day 15 or 20. Group 15 (Fig 9D) peaked on day 15 and dipped on day 20, while subsets peaked on day 1 or 2 or 3. Group 16 (Fig 9D) peaked on days 5 and 15 and dipped on day 10, and subsets dipped on day 1 or 2. Group 17 (Fig 9E) was low at day 0 and peaked on days 1 and 15; a subset peaked on day 5. Group 18 (Fig 9E) peaked on days 0, 5, and 15, dipped on days 10 and 20, and dipped for a subset on day 1. Group 19 (Fig 9E) peaked on day 10 and dipped for subsets on day 1 or 2. Group 20 (Fig 9E) peaked on day 5, and subsets peaked on day 0 or 1.

With biotype H, groups were more populous and more heterogeneous than they were for biotype B. Group 1 (Fig 9F) had subsets that peaked or dipped at each timepoint, although there was a solid majority that peaked on day 2, and expression was generally lower with virus at days 3, 5, 10, and 15. Group 2 (Fig 10A) expression was enhanced on days 3, 15, and 20, and reduced on days 5 or 10. Group 3 (Fig 10B) was mostly elevated on days 5, 15, and 20, and reduced on day 10. Group 4 (Fig 10C) expression was enhanced except on day 5 and for some contigs on day 10. Group 5 (Fig 10C) expression increased from day 10 onward, and the majority of contigs had a minimum on day 5. Group 6 (Fig 10D) peaked on days 0, 10, and 15, and it dipped on days 1, 3, 5, and 20. Group 7 (Fig 10D) expression was elevated on days 1, 2, 3, 15, and 20, and a subset was reduced on days 0, 3, 5, and 10. Group 8 (Fig 10D) peaked on days 0 and 2 and dipped on day 5; some contigs also dipped on days 1 and 10. Group 9 (Fig 10E) peaked on day 10 and dipped on day 0; some of group 9’s contigs also dipped on days 2, 3, or 5. Group 10 (Fig 10E) peaked on days 1, 3, 15, and 20, and dipped on days 2, 5, and 10. Group 11 (Fig 10E) peaked on days 5 and 20 and dipped on days 0 and 15; a subset dipped on days 1, 2, and 3. Group 12 (Fig 10F) mostly peaked on days 0 and 5 and dipped on days 3 and 15. Group 13 (Fig 10F) was enhanced throughout except for a dip on day 5; a minority also dipped on days 0 and 15. Group 14 (Fig 10F) conspicuously dipped on day 5 and less so on days 0 and 15; some members peaked on day 20. Group 15 (Fig 11A) was mostly elevated on days 0, 2, 3, and 5, and it dipped on day 15; individual contigs peaked or dipped on day 20. Group 16 (Fig 11A) was reduced throughout except for a peak on day 15. Group 17 (Fig 11A) was elevated on days 1, 3, 15, and 20, and reduced on days 0 and 5. Group 18 (Fig 11B) alternated peaks on days 0, 2, 5, and 15, with dips on days 1, 3, and 10; a subset dipped on days 15 and 20. Group 19 (Fig 11B) peaked on days 1 and 5 and dipped on day 20. Group 20 (Fig 11B) dipped on days 1, 2, or 3 and peaked on days 0 and 5.

Group 5 in S7 Table lists 12 very significantly and highly expressed proteins (adjusted p-value less than 1e-20, base expression greater than 1000) that were up-regulated up to 60-fold or more in the presence of CYDV across seven timepoints. Two of them map to FK506-binding proteins, a family of peptidylprolylisomerase chaperone proteins that can interact with immunosuppressant drugs to suppress rejection in organ transplant patients [44]. Another one, the most highly expressed of the dozen, is a mitochondrial gene. Two are cytochrome b, and one is a cytochrome c oxidase. Four are ribosomal proteins. Another is homologous to DNAJ1, which affects protein transport into mitochondria (https://www.uniprot.org/uniprotkb/P31689/entry). An RNA helicase and a cold-and-drought regulated protein (possibly a contaminant from wheat) round out the dozen.

### Extrema

Contigs with maximum abs(log_2_(fold-change)) greater than 18 were analyzed separately because they tended to have all expression in one treatment and often in one replicate. While some such contigs represented greenbug genes, others were contaminants from wheat or bacteria. Table 4 summarizes the distribution of contaminating taxa among replicates. S13 Table documents the contaminating genera and gene functions that were detected.

**Table 4:** Distribution of contaminant contigs among replicates and higher taxa.

Replicate	Unannotated	Aphid	Other arthropod	Other animal	Protozoan	Fungus	Wheat	Other plant	Bacterium
B02	3	0	0	0	0	0	1	0	0
B12	0	0	0	0	1	0	1	0	0
B33	0	0	0	0	0	0	0	0	1
B51	3	0	0	0	0	0	0	0	2
BRPV32	84	3	0	0	0	0	307	4	2
BRPV62	2	0	0	0	0	0	0	0	1
BRPV72	8	1	0	0	0	0	1	0	0
H01	15	0	2	0	1	1	0	0	0
H02	3	0	0	1	0	0	2	0	0
H03	11	13	0	0	0	0	1	0	0
H11	1	0	0	0	0	0	1	0	0
H41	18	0	0	0	0	0	12	0	0
H43	6	0	0	0	0	0	4	0	0
HRPV03	1	0	0	0	1	1	0	0	0
HRPV12	1	0	0	1	0	0	1	0	0
HRPV51	7	2	0	0	0	0	3	1	3
HRPV53	31	3	0	0	0	0	14	0	1

Numbers in cells state the count of different contigs to which reads from each replicate were aligned. The replicate codes indicate biotype, collection code, and replicate. Collection codes are 0 for day 0, 1 for day 1, 2 for day 2, 3 for day 3, 4 for day 5, 5 for day 10, 6 for day 15, and 7 for day 20.

Contigs that were expressed in all viral or non-viral replicates at only a single timepoint were sought by examining the counts matrix of each biotype without regard for total read counts. S14 Table gives the examples that were found. Within this set, contigs were sought with expression in all three viral or non-viral replicates. Only three such contigs existed in the dataset, but there were eight more that were represented by two replicates in either the viral or non-viral replicates (Table 5). These 11 were blast-aligned at 1e-04 to both NCBI nr and nt, and the closest identities are given in Table 6. Only six of 11 were attributable to greenbug, while two were unannotated and three were contaminants. The contig that was expressed only in one viral and three non-viral replicates of biotype B on day 10, TRINITY_DN39202_c0_g1_i1, hit accession KAF0767961.1, ras GTPase-activating protein-binding protein 1 from *Aphis craccivora*. The contig that hit three replicates with virus and none without virus on day 15, TRINITY_DN116601_c0_g1_i1, hit accession XP_026811043.1, lysosome membrane protein 2-like from *Rhopalosiphum maidis*. The contig that hit only the three replicates with virus on day 20, TRINITY_DN239434_c0_g1_i1, hit a bacterial amidase.

**Table 5:** Contigs with counts limited to two or more replicates at a single timepoint.

Contig	Day	No virus			With virus		
		Rep1	Rep2	Rep3	Rep1	Rep2	Rep3
TRINITY_DN152059_c0_g1_i1	20	1	16	2	1	30	68
TRINITY_DN239434_c0_g1_i1	20	0	0	0	11	75	15
TRINITY_DN39202_c0_g1_i1	10	59	16	22	0	0	45
TRINITY_DN116601_c0_g1_i1	15	0	0	0	58	25	15
TRINITY_DN194942_c0_g1_i1	0	4	39	0	19	0	54
TRINITY_DN200054_c0_g1_i1	10	13	9	0	77	30	0
TRINITY_DN204733_c0_g1_i1	0	17	0	0	24	0	97
TRINITY_DN21893_c0_g1_i1	10	44	0	5	0	34	224
TRINITY_DN219362_c0_g1_i1	10	14	0	3	0	24	86
TRINITY_DN257075_c0_g1_i1	10	44	25	0	0	3	67
TRINITY_DN94318_c0_g1_i1	5	59	0	89	22	0	0

Each table cell contains a count.

**Table 6:** Identities of closest blast hits for contigs with extremely limited expression.

Contig	Closest blast hit
TRINITY_DN21893_c0_g1_i1	XP_026818447.1 uncharacterized protein LOC113557236 isoform X2 [*Rhopalosiphum maidis*] Length = 936
TRINITY_DN39202_c0_g1_i1	KAF0767961.1 ras GTPase-activating protein-binding protein 1 [*Aphis craccivora*] Length = 371
TRINITY_DN94318_c0_g1_i1	VAI58301.1 unnamed protein product [*Triticum turgidum* subsp. *durum*] Length = 606
TRINITY_DN116601_c0_g1_i1	XP_026811043.1 lysosome membrane protein 2-like [*Rhopalosiphum maidis*] XP_026811044.1 lysosome membrane protein 2-like [*Rhopalosiphum maidis*] XP_026811045.1 lysosome membrane protein 2-like [*Rhopalosiphum maidis*] Length = 556
TRINITY_DN152059_c0_g1_i1	XP_003491361.1 glucose dehydrogenase [FAD, quinone] [*Bombus impatiens*] Length = 610
TRINITY_DN194942_c0_g1_i1	No hit found
TRINITY_DN200054_c0_g1_i1	OU899034.1 *Aphis gossypii* genome assembly, chromosome: 1 Length = 91427719
TRINITY_DN204733_c0_g1_i1	RYG65399.1 hypothetical protein EON64_11980, partial [archaeon] Length = 355
TRINITY_DN219362_c0_g1_i1	OU899035.1 *Aphis gossypii* genome assembly, chromosome: 2 Length = 89130466
TRINITY_DN239434_c0_g1_i1	HCT41739.1 amidase [*Moraxellaceae* bacterium] Length = 491
TRINITY_DN257075_c0_g1_i1	No hit found

### Individual contigs of interest

Since CYDV virions cross the gut lining within lysosomes [45], the expression of contigs involved in lysosome function was examined. There were four such differentially expressed contigs in biotype B and 24 in biotype H. Two functions, lysosomal alpha-mannosidase and lysosomal aspartic protease, were up-regulated with virus in both biotypes, but other isoforms of each were down-regulated in biotype H. Lysosomal Pro-X carboxypeptidase and acid glucosylceramidase were up-regulated in biotype H. Three isoforms of a moderately expressed lysosomal-trafficking regulator were up-regulated 2.7-fold to 3.3-fold in biotype H; all three peaked on day 20.

Cilia et al. [45, 46] reported that peptidylprolylisomerase, a luciferase homolog, and the *Buchnera*-produced symbionin, can bind to CYDV virions and potentially shield them from immune recognition and attack. Peptidylprolylisomerase was differentially expressed as three BH contigs in biotype B and 14 BH contigs in biotype H; two were shared between biotypes. The four with greatest base expression, all significant only in H, were up- or down-regulated less than two-fold. The other 11 were up-regulated two- to 540-fold with virus. Ten peaked on day 20, four peaked on day 15, and one peaked on day 2. None of the differentially expressed contigs was annotated as luciferase-like or as symbionin.

Amino-acid transporters were also examined, since aphids have unusually large families of amino-acid transporters that move amino acids into aphid cytoplasm from the intracellular, endosymbiotic *Buchnera aphidicola* bacteria that remedy aphids’ severely deficient diet of plant sap [47]. There were 17 differentially expressed BH amino-acid transporter contigs in biotype B and 54 in biotype H. Only six were in common, implying the existence of many other isoforms that were not differentially expressed. In biotype B, the extremes were down-regulated 310-fold to up-regulated 225-fold with virus, but 10 were changed less than five-fold and 11 were up-regulated. In biotype H, six were down-regulated up to 12-fold, 36 were up-regulated less than 40-fold, and 12 were up-regulated 44- to 310-fold. Peak dates were 2 for 19 contigs, 15 for 17 contigs, and 20 for 16 contigs.

Seventeen distinct, putative aphid or *Buchnera* effectors were sought out in the results (Table 1). Symbionin, produced by *Buchnera aphidicola*, was not found among the expression counts and was likely stripped out as a contaminant before analysis. Two of the aphid effectors, JX134487.1 and NP_001313555.1, were significantly up-regulated with virus in biotype H. Nine other closely related effectors mapped with NP_001313555.1.

Contigs with significant differential expression for at least one timepoint were compared to the top-20 lists of differentially up- and down-expressed proteins in two cryptic species in *Bemisia tabaci* (Gennadius) *s*.*l*. as compiled by Kliot et al. [48]. *Bemisia tabaci* (the sweet potato whitefly) belongs to the same suborder of Hemiptera as aphids. The results appear in S15 Table, and a summary appears in Table 7. The top-20 lists of the Mediterranean and Asia Minor cryptic species in *Bemisia tabaci* overlap enough that the total number of distinct proteins is 58 instead of 80. Only about 25 to 35% of biotype B and biotype H contigs share possible or certain functionality with the *Bemisia* proteins, as based on the names of the proteins.

**Table 7:** Counts of significantly differentially expressed contigs in biotypes B and H that appeared in top-20 lists of differential expression in *Bemisia tabaci*.

Set	Found	Fraction found	Possibly found	Fraction possibly found	Not found	Fraction not found
B-KSU	5	0.086	10	0.172	43	0.741
B-BH	5	0.086	12	0.207	41	0.707
H-KSU	5	0.086	9	0.155	44	0.759
H-BH	6	0.103	14	0.241	38	0.655
MED	22	0.379	0	0.000	36	0.621
MEAM	37	0.638	0	0.000	21	0.362

Sets are B-KSU, biotype B with KSU transcriptome; B-BH, biotype b with BH transcriptome; H-KSU, biotype H with KSU transcriptome; H-BH, biotype H with BH transcriptome; MED, Mediterranean cryptic species in *B. tabaci s.l.*; MEAM, Middle Eastern-Asia Minor cryptic species in *B. tabaci s.l*. MED and MEAM are calculated from Figs 2 and 3 in Kliot et al. [48].

## Discussion

### General

This study was designed to detect and describe changes in greenbug gene expression in response to a developing CYDV infection in host wheat plants. Uninfected wheat served as a control that was also subject to aphid feeding. The experiment followed a full factorial design with factors time, biotype, and acquired CYDV. Two biotypes were examined for difference and commonality in altered gene expression. The sampling covered a substantial fraction of the lifespan of individual aphids and a substantial period of disease progression on the wheat. The protocol for collecting mRNA was intended to minimize handling of and injury to the greenbugs before flash-freezing them. A second goal was to search for direct effects of CYDV on gene expression in its carrier aphids. By placing viruliferous or aviruliferous greenbugs on uninfected wheat, the differentiating effects of diet, population density, and wheat defense response were negated at the early timepoints.

### Assembly quality and contamination

Two Trinity transcriptome assemblies were used for counting cDNA reads to genes. The first, under Genbank master record GIML00000000.1, was from a different conspecific biotype, but it had the appearance of a good transcriptome assembly with a plausible number of expressed genes (23527) and reasonable N50 of 2823. The second, produced from a sample of 94.2 gigabases of reads from the current study itself, was implausibly large (294464 contigs before removal of obvious contaminants) and had a lower N50 of 1616. Busco analysis indicated that both assemblies were 97–99% complete and neither was particularly fragmented.

An aphid is an ecosystem of insect, gut and surficial microflora, and intracellular endosymbionts including but not limited to *Buchnera aphidicola* [49]. It is not feasible to separate these organisms when extracting RNA from the whole body. Therefore, both genomic and transcriptomic assemblies must deal with contamination of the read set with microbes on or within the body of the aphid. Furthermore, the flash-freezing of aphids on a piece of wheat leaf, followed by removing the aphids to extraction buffer, can introduce wheat and microbial contamination from the leaf tissue and the feeding site. This contamination spans the wheat and microbial genomes, rendering rRNA sequence databases useless for purging contaminating reads and contigs assembled from them. Alignment with blastn to whole bacterial, archaeal, fungal, and protist genomes in NCBI databases allowed the removal of contigs from sequenced organisms, but that could not remedy contamination from unsequenced organisms. The continued presence of such contaminant contigs contributed some unknown amount to the excessive number of contigs in the BH assembly. On the other hand, mapping the BH transcriptome assembly to the Kansas Great Plain greenbug genome implicitly assumed that the Kansas Great Plain assembly was complete and free of collapsed paralogs, and it still retained 177199 contigs. It is likely that the contig count was increased by the relatively high frequency of multiple isoforms resulting from alternative splicing.

Another unsettling difference in comparing results between the KSU and BH transcriptomes was the use of different software to align reads to the reference contigs. STAR was used with the KSU transcriptome, but the BH transcriptome was too large for STAR indexing to be feasible. This necessitated indexing and alignment of BH with bwa mem. Within the available computational resources, it was assumed that STAR and bwa mem produced equivalent alignments and resulted in equivalent hit counts.

### Results of likelihood ratio tests

Likelihood ratio tests can be used with DESeq2 to investigate the relative importance of different factors in controlling gene expression. Such tests involve pairs of statistical models where one model is a subset (reduced version) of the other model. If the two models identify no differentially expressed genes or isoforms, one can conclude that the extra factor(s) in the complete model did not affect expression. It was assumed that the importance of a factor (virus, biotype, time, or a combination or interaction) was monotonically related to the fraction of significantly differentially expressed genes when models were compared with and without the factor. Therefore, the results in Table 2 confirmed that virus had much less effect on gene expression than time or biotype in comparison to the KSU transcriptome, but about equal effect with time or biotype in comparison with the BH transcriptome. It is not obvious why the transcriptomes produced such different results.

### Day of most deviant expression

The time course was analyzed by comparing log_2_ fold-change with virus to no virus at each timepoint and then comparing those pairwise comparisons among the timepoints. In consequence, there was a day for each contig where abs(log_2_ fold-change) was maximal, implying that differential expression was maximal. Thus early-responding contigs could be separated from late-responding contigs. The early-responding contigs were considered more likely to represent a direct response to CYDV, while the late-responding contigs were more likely to represent a response to a diseased host or high population density or a shift to the alate form. The high fraction of late-responding contigs was consistent with a shift to alate life form in response to starvation on moribund wheat in the presence of CYDV. Day 0 differed from the others in that the viruliferous aphids were on the maintenance wheat plants that had CYD symptoms, whereas the viruliferous aphids were on initially uninfected wheat from day 1 onward and responded to the initially healthy wheat host.

Contigs whose differential expression (abs(log_2_ fold-change)) peaked on day 1 were of interest as possibly responding directly to CYDV acquisition, because the wheat host had had little time to respond to the virus. Of the top 100 contigs top-ranked by adjusted p-value on day 1 in biotype B, 69 have their top ranking at day 15 or 20 in biotype H, which is consistent with 83% of contigs at large having top ranking on day 15 or 20. There were 59 completely unannotated contigs and 10 uncharacterized or unnamed contigs among these top 100. One of these proteins, XP_026815011.1, ATP-dependent RNA helicase p62, which was upregulated 30-60-fold with CYDV, is thought to have a role in innate immunity (https://www.uniprot.org/uniprotkb/P19109/entry) [50] as well as regulating transcription and alternative splicing. Upregulation of an antiviral activity suggests that the greenbug immune system was detecting CYDV, even if viral RNA was never released from the virions. At least five other potentially regulatory proteins were also among the top 100 on day 1. Four of them were up-regulated at least 20-fold with CYDV: XP_003241030.1 (histone H2B-like), XP_026816583.1 (Bloom syndrome protein homolog), XP_025190740.1 (protein stunted-like), and XP_026818026.1 (cyclin-K). The fifth protein, XP_026816116.1 (proton-coupled amino acid transporter-like protein pathetic), was down-regulated 284-fold in biotype B but only 4.3-fold in biotype H.

There were four other RNA helicases among contigs that were differentially expressed in both biotypes B and H over the time course. Three of them, all mapping to ATP-dependent RNA helicase p62, were up-regulated at least 97-fold at peak response: XP_026815011.1, XP_025199436.1, and XP_026807159.1. The fourth, XP_026822389.1 (probable ATP-dependent RNA helicase DDX43), was up- or down-regulated less than two-fold in both biotypes. All five reached maximal differential expression at day 20 in at least one biotype (S16 Table).

### Hierarchical clustering

The values of log_2_ fold-change by date comprised an expression profile, and distances (Euclidean or otherwise) could be calculated among expression profiles. Hierarchical clustering is one of the traditional ways to recognize natural groups among expression profiles of contigs that were differentially expressed for at least one timepoint. However, most contigs were not differentially expressed, and hierarchical clustering tended to peel off small groups of contigs with distinctive expression profiles while leaving unresolved a larger, cohesive set of less differentially expressed contigs with more subtly divergent expression profiles. The numerical values in expression profiles were complicated by the intrinsic noisiness of read counts to contigs, which was affected not only by sampling error but also population age structure and individual variation in microenvironment and life history.

The choice of 20 subclusters was arbitrary and represented a compromise between human comprehension and homogeneity within subclusters. Even with 20 subclusters, most subclusters were heterogeneous as evidenced by scattering among curves in Figs 7–11. Also, there was little or no functional relationship among the top-ranking contigs within each subcluster, although a formal network analysis might have found some relationships.

An alternative to hierarchical clustering would have been classification versus standard profiles with some value to distinguish “up” from “equal” from “down” at each timepoint. Classification is generally effective to divide a large, cohesive but heterogeneous cluster into meaningful groups. A given expression profile could be mapped to the closest standard profile by Euclidean distance after normalization to account for count totals. The problem with this approach is the large number of possible standard profiles, e.g., 3^8^ = 6561 for eight timepoints and three levels per timepoint. A second alternative would have been a self-organizing map [51], but again any tractable number of cells in the map would entail high heterogeneity within cells.

### Differences between biotypes

As mentioned above, biotypes B and H differed in response for specific contigs. There were also pervasive differences across the entire set of differentially expressed contigs. More contigs responded in biotype H than in biotype B. For alignments to the KSU transcriptome, there were 328 contigs that had significant p-values for biotype B only, 712 that were significant for biotype H only, and 66 that were significant for both. For alignments to the BH transcriptome, 2814 contigs were significant for B only, 14985 were significant for H only, and 1826 were significant in both. The relatively low number of contigs responding in both biotypes could arise because the two biotypes differ in how they respond to acquired virus or infected wheat, or because only relatively few contigs are involved in the response to virus and the others are responding to something else that was uncontrolled despite all efforts to maintain a uniform environment. The former seems more plausible.

### Noteworthy genes

Aphids have unusually large families of amino-acid transporters that move amino acids into aphid cytoplasm from the intracellular, endosymbiotic *Buchnera aphidicola* bacteria that remedy aphids’ severely deficient diet of plant sap [47]. While some amino-acid transporters were down-regulated with CYDV, most were up-regulated and some were up-regulated strongly. The physical location of the different transporters would matter, since CYDV has been shown to increase the amino-acid content of wheat sap at least transiently [19], which could alter the balance between the gut and bacteriocytes in supplying amino acids elsewhere in the aphid body.

A positive feedback between viral load and peptidylprolylisomerase activity would benefit CYDV if in fact peptidylprolylisomerase can protect CYDV virions. However, the observed general up-regulation of peptidylprolylisomerase with virus does not confirm that such a feedback loop exists.

Effector proteins can thwart wheat defense response and increase aphid weight and fecundity [41]. The decreased expression of candidate effectors in biotype B and increased expression in biotype H with acquired virus implies that viruliferous biotype B would be less successful than aviruliferous B on Newton wheat, while viruliferous biotype H would be more successful. Much would depend on the relative effectiveness of the two significantly up-regulated effectors versus the other effectors.

While biotype B effectively transmitted CYDV-RPV to wheat in this study, biotype B failed to transmit CYDV-RPV to oats in a study by Tamborindeguy et al. [14], who used a 48-hour acquisition access period versus our 13-day acquisition access period. Biotype H effectively transmitted this virus in both studies.

Aphids have a reduced immune system, perhaps as an adaptation to their intracellular endosymbionts and their relatively sterile diet of plant sap. Several major classes of insect defense peptides and signaling proteins have been lost in the evolution of aphids [52]. What remains of aphid antimicrobial immunity acts through melanization, reactive oxygen species, and hemocyte-mediated phagocytosis [53], and the Jun N-terminal kinase is important in activating this response by phosphorylating transcription factors. Less seems to be known about antiviral immunity in aphids. However, cyclophilin and a luciferase homolog have been found to bind CYDV-RPV virions in greenbug [46], and up to five-fold increased expression of cyclophilin (referenced as peptidylprolylisomerase) was correlated with increased transmission of RPV to wheat in an F_2_ greenbug population segregating for transmission competence [43]. In biotype H, there were 14 differentially expressed BH contigs for peptidylprolylisomerase. All but one were maximally expressed on days 15 or 20, and three were up-regulated more than 200-fold. Only three others were down-regulated. In biotype B, there were three differentially expressed BH peptidylprolylisomerase contigs, and all were up-regulated more than 170-fold. Thus the current study adds to the evidence that peptidylprolylisomerase facilitates transmission of CYDV. In contrast, only two significant biotype-H BH contigs were annotated as processing luciferin: luciferin sulfotransferase-like and luciferin 4-monooxygenase-like. The latter was down-regulated 150-fold with virus on day 0.

The relationship of cuticle proteins to CYDV acquisition is complicated. CYDV is acquired through the aphid hindgut [46], the insect hindgut is lined with a thin cuticle [54], and decreased expression of cuticular proteins in the hindgut might render it more permeable to virions. On the other hand, cuticular proteins are produced during molting of instars, so increased expression of cuticular proteins could reflect increased fecundity and a shift of population structure to immature stages. It is even possible that the hindgut is particularly permeable during molting, so that decreased cuticular expression could increase viral uptake then. Another consideration is the balance among cuticular constituents; cuticles vary in stiffness and consist of mixtures of chitin, proteins, and waxes [54], with great potential for variation among the proteins. It is not surprising then that biotype H had 103 significantly differentially expressed BH contigs for cuticular proteins, of which 18 had a minimum adjusted p-value less than 1e-6. Only seven of these had decreased expression with acquired virus. The generally increased expression is consistent with the hypothesis that virus increased greenbug fecundity over the sampled time course. However, the maximum expression was on day 2 or 3 for 78 of these contigs, opening the possibilities that the aphids were mounting a transient defense response to the virus or responding to the wheat’s defense response to the virus or briefly slowing their maturation.

In conclusion, time is important in understanding the interactions among greenbugs, wheat, and CYDV-RPV. There is an interval where infection might improve the nutritional quality of wheat sap as the greenbug’s diet, but ultimately the wheat succumbs to virus and aphid attack, and dead wheat does not support aphids. Early during infestation, the aphid population expectedly follows a logistic growth curve, but the population inevitably declines later as the plant dies and aphids starve or move on to other plants. Conclusions about viral effects on carrier aphid fecundity or dry weight must take time into account. Also, as shown here, aphid genotype and time shape aphid gene expression and response to carried virus and an infected host plant. The influence of an infected host increases with time.

## Supporting information

S1 Raw images (PDF)

S1 Table Virus-specific and host-specific PCR primers. (DOCX)

S2 Table RNA sample statistics. (DOCX)

S3 Table Biotype B contigs whose expression differential peaked at each timepoint. (XLSX)

S4 Table Biotype H contigs whose expression differential peaked at each timepoint. (XLSX)

S5 Table Roster of KSU expression profile groups for biotype B. (XLSX)

S6 Table Roster of KSU expression profile groups for biotype H. (XLSX)

S7 Table Roster of BH expression profile groups for biotype B. (XLSX)

S8 Table Roster of BH expression profile groups for biotype H. (XLSX)

S9 Table Summary of contig expression patterns for biotype B and the KSU transcriptome. (DOCX)

S10 Table Summary of contig expression patterns for biotype H and the KSU transcriptome. (DOCX)

S11 Table Summary of contig expression patterns for biotype B and the BH transcriptome. (DOCX)

S12 Table Summary of contig expression patterns for biotype H and the BH transcriptome. (DOCX)

S13 Table Functional annotation of contigs with extremely restricted expression. (XLSX)

S14 Table Examples of extremely restricted expression. (XLSX)

S15 Table Comparison of most differential expression in *Bemisia tabaci* to differential expression in biotypes B and H. (DOCX)

S16 Table Contigs that are differentially expressed in both biotypes, arranged by adjusted p-value in biotype B. (XLSX)

## Abbreviations

AAP: acquisition access period
BH: biotypes B and H
BYDV: barley yellow-dwarf virus
CP: coat protein
CYDV: cereal yellow-dwarf virus
FDR: false discovery rate
IAP: inoculation access period
KSU: Kansas State University
MAV: *Luteovirus mavhordei*
NCBI: National Center for Biotechnology Information
PAV: *Padi avenae* virus
RMV: ribgrass mosaic virus
RPV: *Rhopalosiphum padi* virus
SBWMV: soil-borne wheat mosaic virus
SGV: *Schizaphis graminum* virus
trnL: transfer RNA for leucine
USDA-ARS: United States Department of Agriculture, Agricultural Research Service
WSMV: wheat streak mosaic virus
WSSMV: wheat spindle streak mosaic virus
