# Supplementary figures and images for: Differential gene expression between viruliferous and non-viruliferous Schizaphis graminum (Rondani)
Source: PLoS One. 2023 Nov 8;18(11):e0294013. doi: 10.1371/journal.pone.0294013 (PMC10631655; doi:10.1371/journal.pone.0294013)

Fig. 1

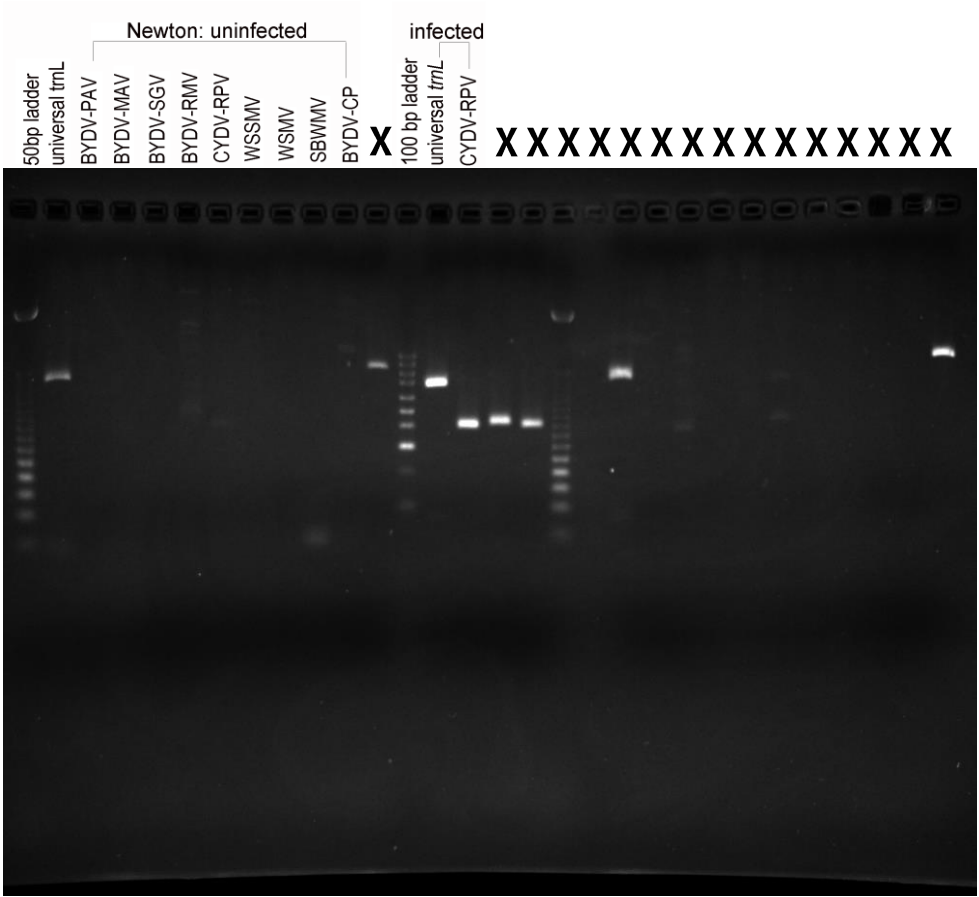

Fig. 2

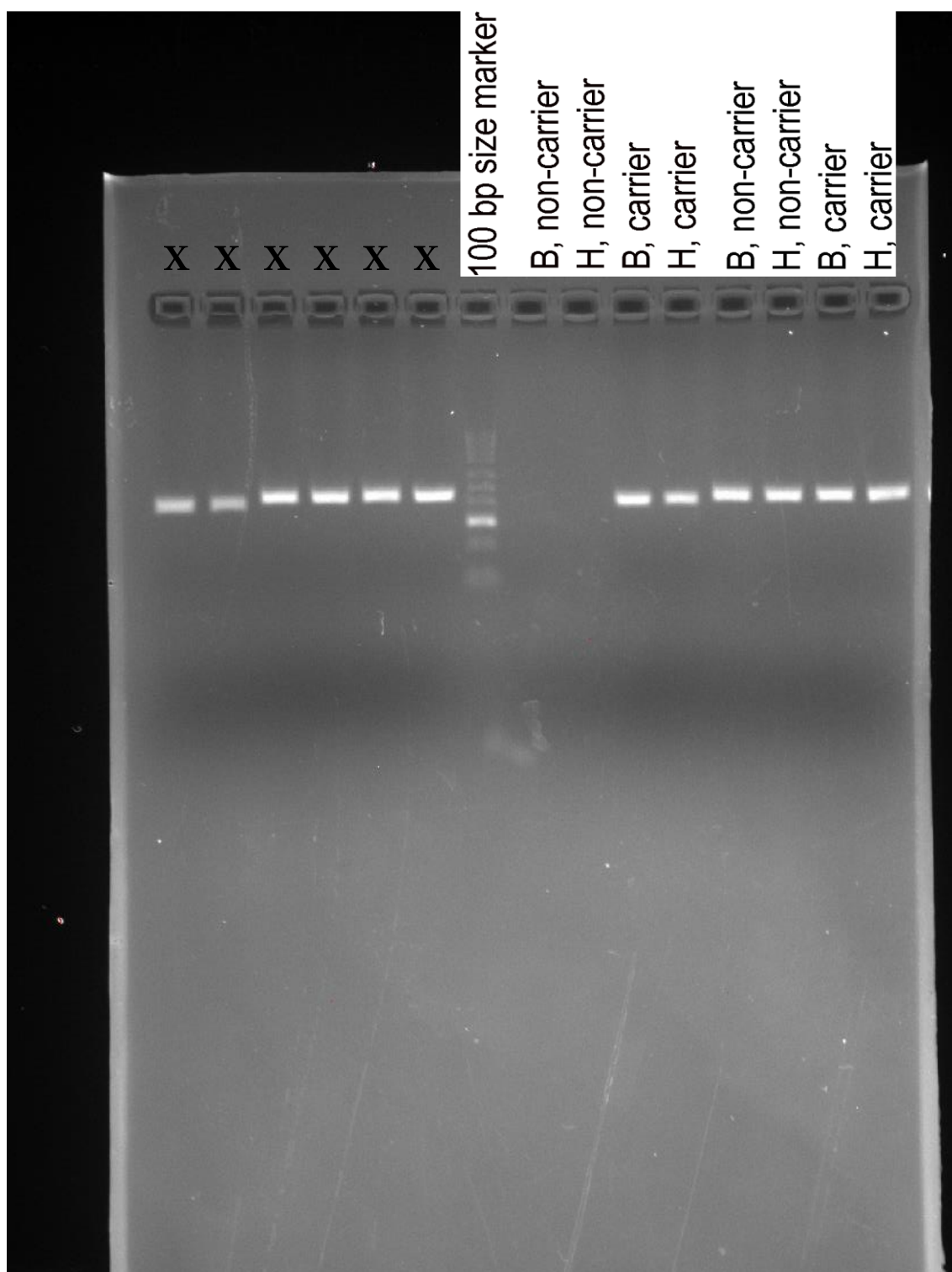

Fig. 3

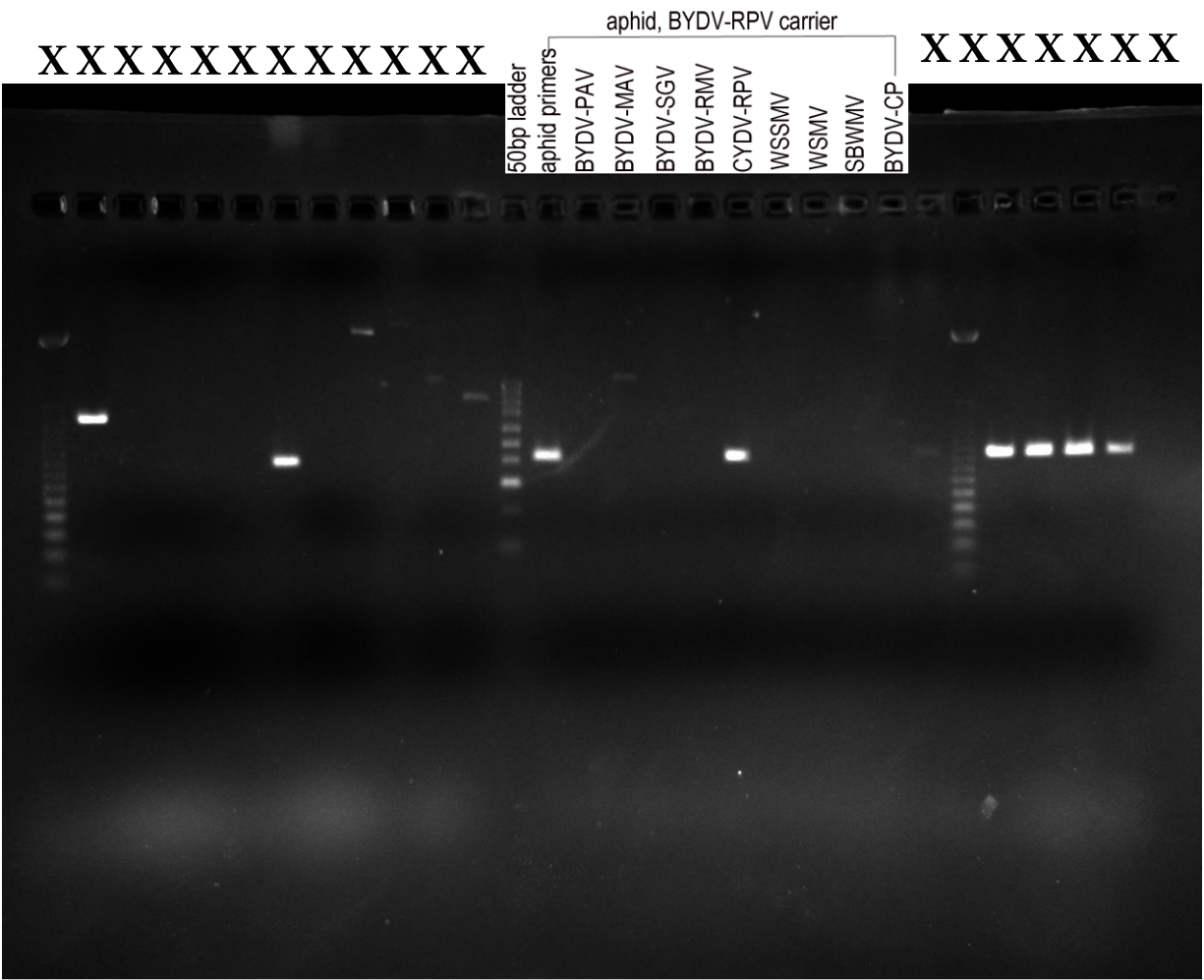

Supplement: S1 Raw images — (PDF) [file pone.0294013.s001.pdf]
