# Supplementary material for: Differential gene expression between viruliferous and non-viruliferous Schizaphis graminum (Rondani)
Source: PLoS One. 2023 Nov 8;18(11):e0294013. doi: 10.1371/journal.pone.0294013 (PMC10631655; doi:10.1371/journal.pone.0294013)
Supplement: S1 Table — (DOCX) [file pone.0294013.s002.docx]

| **ID** | **Forward primer** | **Tm (^0^C)** | **Reverse primer** | ***Tm* (^0^C)** | **Amplicon size (bp)** | **NCBI source** |
| --- | --- | --- | --- | --- | --- | --- |
| **BYDV-PAV** | AGAGGAGGGGCAAATCCTGT | 59.4 | ATTGTGAAGGAATTAATGTA | 47.1 | 295 | D11032 |
| **BYDV-MAV** | CAACGCTTAACGCAGATGAA | 55.3 | AGGACTCTGCAGCACCATCT | 59.4 | 175 | D11028 |
| **BYDV-SGV** | ACCAGATCTTAGCCGGGTTT | 57.3 | CTGGACGTCGACCATTTCTT | 57.3 | 237 | AY541039.1 |
| **BYDV-RMV** | GACGAGGACGACGACCAAGTGGA | 66.0 | GCCATACTCCACCTCCGATT | 59.4 | 365 | L12757.1 |
| **CYDV-RPV** | ATGTTGTACCGCTTGATCCAC | 57.9 | GCGAACCATTGCCATTG | 52.8 | 400 | AF235168.2 |
| **WSSMV** | GCAACCCTTAGCGAAGTCAG | 59.4 | GAGGCTCCGTGTCTCATAGC | 61.4 | 154 | X73883 |
| **WSMV** | CGACAATCAGCAAGAGACCA | 57.3 | TGAGGATCGCTGTGTTTCAG | 57.3 | 193 | NC 001886 |
| **SBWMV** | CCTATGGCGTCCTAACGTGT | 59.4 | CACAATCTGCAGGAAGACGA | 57.3 | 219 | NC 002042 219 |
| **BYDV-CP** | TTCAGTAGGCCGTAGAGGAC | 62.2 | TTGTGATCTTGTAACGGTGG | 59.7 | 499 |  |
| **Aphid** | CCCAGCAGGAGGAGGTGA | 60.3 | CACCTGTTAGCCCTCCAATTGT | 60.6 | 404 | AF285905.1 |
| **universal trnL1** | CGAAATCGGTAGACGCTACG | 62.0 | GGGGATAGAGGGACTTGAAC | 60.8 |  |  |
| **universal tmL2** | AAAGTGGGTTTTTATGATCC | 56.9 | TTAAAAGCCGAGTACTCTACC | 60.0 |  |  |
