# Supplementary material for: Differential gene expression between viruliferous and non-viruliferous Schizaphis graminum (Rondani)
Source: PLoS One. 2023 Nov 8;18(11):e0294013. doi: 10.1371/journal.pone.0294013 (PMC10631655; doi:10.1371/journal.pone.0294013)
Supplement: S2 Table — (DOCX) [file pone.0294013.s003.docx]

| ID^a^ | Total Gb | rRNA % | Clean Data Gb |
| --- | --- | --- | --- |
| B01 | 27.3 | 31.97 | 18.57 |
| B02 | 16.1 | 65.53 | 5.55 |
| B03 | 20.9 | 24.72 | 15.73 |
| B11 | 17 | 42.31 | 9.81 |
| B12 | 20.1 | 40.35 | 11.99 |
| B13 | 20.1 | 17.87 | 16.51 |
| B21 | 27.7 | 61.91 | 10.55 |
| B22 | 27.5 | 75.05 | 6.86 |
| B23 | 29.3 | 64.12 | 10.51 |
| B31 | 24.5 | 48.41 | 12.64 |
| B32 | 26 | 55.93 | 11.46 |
| B33 | 25.4 | 27.06 | 18.53 |
| B41 | 11.9 | 55.68 | 5.27 |
| B42 | 22.3 | 46.77 | 11.87 |
| B43 | 25.8 | 56.98 | 11.10 |
| B51 | 22.3 | 43.46 | 12.61 |
| B52 | 10.7 | 43.08 | 6.09 |
| B53 | 22.6 | 50.07 | 11.28 |
| B61 | 11.8 | 42.29 | 6.81 |
| B63 | 10.1 | 39.62 | 6.10 |
| B71 | 8.9 | 25.02 | 6.67 |
| B72 | 8.3 | 20.60 | 6.59 |
| B73 | 8.9 | 26.77 | 6.52 |
| H01 | 27.6 | 27.54 | 20.00 |
| H02 | 26.7 | 35.91 | 17.11 |
| H03 | 20.8 | 75.43 | 5.11 |
| H11 | 24.2 | 25.33 | 18.07 |
| H12 | 20.9 | 36.78 | 13.21 |
| H13 | 20.3 | 20.43 | 16.15 |
| H21 | 23.3 | 46.50 | 12.46 |
| H22 | 26.8 | 32.72 | 18.03 |
| H23 | 21.4 | 43.19 | 12.16 |
| H31 | 27.4 | 20.45 | 21.80 |
| H32 | 23 | 24.04 | 17.47 |
| H33 | 25.1 | 21.69 | 19.66 |
| H41 | 23.7 | 45.08 | 13.02 |
| H42 | 21.9 | 52.47 | 10.41 |
| H43 | 20.5 | 54.51 | 9.33 |
| H51 | 21.6 | 26.96 | 15.78 |
| H52 | 23.6 | 46.00 | 12.74 |
| H53 | 22.4 | 20.88 | 17.72 |
| H61 | 8.9 | 23.37 | 6.82 |
| H62 | 7.1 | 21.79 | 5.55 |
| H63 | 8.1 | 23.81 | 6.17 |
| H71 | 7.5 | 15.00 | 6.38 |
| H72 | 7.5 | 27.98 | 5.40 |
| H73 | 8.3 | 24.52 | 6.26 |
| BRPV01 | 23.5 | 16.15 | 19.70 |
| BRPV02 | 30.3 | 67.72 | 9.78 |
| BRPV03 | 22 | 42.47 | 12.66 |
| BRPV11 | 22.3 | 52.02 | 10.70 |
| BRPV12 | 27.4 | 49.02 | 13.97 |
| BRPV13 | 61.5 | 88.47 | 7.09 |
| BRPV21 | 27.7 | 71.08 | 8.01 |
| BRPV22 | 24.7 | 51.60 | 11.95 |
| BRPV23 | 22.5 | 52.84 | 10.61 |
| BRPV31 | 19.7 | 62.64 | 7.36 |
| BRPV32 | 20 | 27.12 | 14.58 |
| BRPV33 | 21.9 | 54.10 | 10.05 |
| BRPV41 | 18.2 | 37.25 | 11.42 |
| BRPV42 | 17.6 | 62.67 | 6.57 |
| BRPV43 | 19.6 | 70.00 | 5.88 |
| BRPV51 | 22.7 | 57.74 | 9.59 |
| BRPV52 | 12 | 54.37 | 5.48 |
| BRPV53 | 29.8 | 27.26 | 21.68 |
| BRPV61 | 11 | 39.21 | 6.69 |
| BRPV62 | 11.2 | 21.90 | 8.75 |
| BRPV63 | 9.7 | 41.35 | 5.69 |
| BRPV71 | 7.9 | 24.91 | 5.93 |
| BRPV72 | 10.2 | 35.50 | 6.58 |
| BRPV73 | 9.7 | 32.63 | 6.53 |
| HRPV01 | 25.9 | 63.11 | 9.56 |
| HRPV02 | 10.5 | 46.51 | 5.62 |
| HRPV03 | 28.5 | 51.64 | 13.78 |
| HRPV11 | 11.5 | 50.98 | 5.64 |
| HRPV12 | 18.6 | 62.20 | 7.03 |
| HRPV13 | 24.7 | 47.71 | 12.92 |
| HRPV21 | 21.2 | 47.24 | 11.18 |
| HRPV22 | 20.4 | 39.73 | 12.30 |
| HRPV23 | 21.8 | 47.71 | 11.40 |
| HRPV31 | 20.7 | 40.21 | 12.38 |
| HRPV32 | 25.7 | 68.10 | 8.20 |
| HRPV33 | 22 | 43.94 | 12.33 |
| HRPV41 | 25.02 | 77.14 | 5.72 |
| HRPV42 | 10.4 | 41.58 | 6.08 |
| HRPV43 | 17.1 | 65.50 | 5.90 |
| HRPV51 | 17.6 | 65.40 | 6.09 |
| HRPV52 | 23 | 41.51 | 13.45 |
| HRPV53 | 27.3 | 30.93 | 18.86 |
| HRPV61 | 9.7 | 28.33 | 6.95 |
| HRPV62 | 9 | 26.31 | 6.63 |
| HRPV63 | 8.5 | 28.43 | 6.08 |
| HRPV71 | 8.1 | 27.03 | 5.91 |
| HRPV72 | 8.3 | 27.13 | 6.05 |
| HRPV73 | 8.1 | 21.55 | 6.35 |
| Total | 1840.82 |  | 1000.09 |

Supplemental Table 1. RNA sample statistics.

^a^First letter of ID represents the biotype, B or H; RPV represents presence of CYDV-RPV; digits represent the timepoint (1 through 7) and replicate (1 through 3).
