# Supplementary material for: Differential gene expression between viruliferous and non-viruliferous Schizaphis graminum (Rondani)
Source: PLoS One. 2023 Nov 8;18(11):e0294013. doi: 10.1371/journal.pone.0294013 (PMC10631655; doi:10.1371/journal.pone.0294013)
Supplement: S9 Table — (DOCX) [file pone.0294013.s010.docx]

| Group | **Total** | **MinDate** | **MinFrac** | **MaxDate** | **MaxFrac** |
| --- | --- | --- | --- | --- | --- |
| 1 | 46 | 1 | 0.870 | 10 | 0.261 |
| 2 | 194 | 20 | 0.247 | 5 | 0.351 |
| 3 | 23 | 2 | 0.826 | 3 | 0.696 |
| 4 | 5 | 2 | 0.800 | 10 | 0.400 |
| 5 | 14 | 20 | 0.571 | 3 | 0.643 |
| 6 | 9 | 20 | 0.556 | 5 | 0.556 |
| 7 | 19 | 2 | 0.368 | 5 | 1.000 |
| 8 | 4 | 15 | 1.000 | 0 | 0.250 |
| 9 | 2 | 1 | 1.000 | 10 | 1.000 |
| 10 | 5 | 2 | 0.400 | 1 | 0.800 |
| 11 | 8 | 3 | 0.375 | 10 | 0.625 |
| 12 | 2 | 10 | 1.000 | 2 | 1.000 |
| 13 | 20 | 2 | 0.300 | 3 | 0.850 |
| 14 | 11 | 20 | 1.000 | 5 | 0.273 |
| 15 | 3 | 3 | 0.333 | 2 | 0.667 |
| 16 | 4 | 20 | 1.000 | 3 | 0.500 |
| 17 | 14 | 1 | 0.214 | 15 | 0.857 |
| 18 | 4 | 20 | 0.500 | 0 | 1.000 |
| 19 | 4 | 5 | 1.000 | 0 | 0.250 |
| 20 | 2 | 2 | 1.000 | 10 | 1.000 |

Total, count of contigs in the cluster; MinDate, timepoint with the most minimum values of log_2_ fold-change; MinFrac, fraction of contigs having the MinDate timepoint; MaxDate, timepoint with the most maximum values of log_2_ fold-change; MaxFrac, fraction of contigs having the MaxDate timepoint.
