# Supplementary material for: Differential gene expression between viruliferous and non-viruliferous Schizaphis graminum (Rondani)
Source: PLoS One. 2023 Nov 8;18(11):e0294013. doi: 10.1371/journal.pone.0294013 (PMC10631655; doi:10.1371/journal.pone.0294013)
Supplement: S10 Table — (DOCX) [file pone.0294013.s011.docx]

| **Group** | **Total** | **MinDate** | **MinFrac** | **MaxDate** | **MaxFrac** |
| --- | --- | --- | --- | --- | --- |
| **1** | 547 | 10 | 0.176 | 2 | 0.364 |
| **2** | 50 | 1 | 0.360 | 2 | 0.980 |
| **3** | 20 | 5 | 0.550 | 2 | 0.400 |
| **4** | 23 | 5 | 0.391 | 0 | 1.000 |
| **5** | 3 | 0 | 0.333 | 2 | 0.667 |
| **6** | 22 | 10 | 0.364 | 2 | 0.545 |
| **7** | 5 | 1 | 0.400 | 5 | 0.800 |
| **8** | 2 | 3 | 0.500 | 0 | 1.000 |
| **9** | 11 | 0 | 0.273 | 15 | 0.727 |
| **10** | 17 | 5 | 0.941 | 2 | 0.294 |
| **11** | 14 | 15 | 0.714 | 5 | 0.500 |
| **12** | 27 | 10 | 0.259 | 5 | 0.815 |
| **13** | 3 | 0 | 0.667 | 1 | 0.333 |
| **14** | 7 | 1 | 0.429 | 0 | 0.571 |
| **15** | 4 | 0 | 1.000 | 3 | 0.250 |
| **16** | 2 | 0 | 1.000 | 5 | 1.000 |
| **17** | 4 | 5 | 0.500 | 3 | 1.000 |
| **18** | 5 | 5 | 0.600 | 2 | 1.000 |
| **19** | 7 | 15 | 0.714 | 3 | 0.429 |
| **20** | 4 | 3 | 1.000 | 5 | 0.750 |

Total, count of contigs in the cluster; MinDate, timepoint with the most minimum values of log_2_ fold-change; MinFrac, fraction of contigs having the MinDate timepoint; MaxDate, timepoint with the most maximum values of log_2_ fold-change; MaxFrac, fraction of contigs having the MaxDate timepoint.
