# Supplementary material for: Differential gene expression between viruliferous and non-viruliferous Schizaphis graminum (Rondani)
Source: PLoS One. 2023 Nov 8;18(11):e0294013. doi: 10.1371/journal.pone.0294013 (PMC10631655; doi:10.1371/journal.pone.0294013)
Supplement: S11 Table — (DOCX) [file pone.0294013.s012.docx]

| **Cluster** | **Total** | **MinDate** | **MinFrac** | **MaxDate** | **MaxFrac** |
| --- | --- | --- | --- | --- | --- |
| **1** | 316 | 1 | 0.759 | 5 | 0.253 |
| **2** | 1670 | 20 | 0.224 | 5 | 0.253 |
| **3** | 218 | 2 | 0.431 | 15 | 0.546 |
| **4** | 323 | 0 | 0.378 | 20 | 0.356 |
| **5** | 732 | 0 | 0.645 | 15 | 0.296 |
| **6** | 85 | 1 | 0.600 | 2 | 0.471 |
| **7** | 30 | 0 | 0.367 | 1 | 0.333 |
| **8** | 581 | 20 | 0.487 | 3 | 0.420 |
| **9** | 65 | 5 | 0.231 | 20 | 0.800 |
| **10** | 95 | 5 | 0.442 | 15 | 0.326 |
| **11** | 58 | 2 | 0.293 | 3 | 0.759 |
| **12** | 53 | 15 | 0.358 | 1 | 0.736 |
| **13** | 16 | 2 | 0.500 | 1 | 0.562 |
| **14** | 28 | 20 | 0.321 | 2 | 0.286 |
| **15** | 119 | 5 | 0.294 | 1 | 0.529 |
| **16** | 115 | 1 | 0.730 | 15 | 0.557 |
| **17** | 39 | 0 | 0.385 | 1 | 0.333 |
| **18** | 60 | 20 | 0.517 | 5 | 0.717 |
| **19** | 32 | 2 | 0.469 | 10 | 0.469 |
| **20** | 5 | 20 | 0.600 | 0 | 0.400 |

Total, count of contigs in the cluster; MinDate, timepoint with the most minimum values of log_2_ fold-change; MinFrac, fraction of contigs having the MinDate timepoint; MaxDate, timepoint with the most maximum values of log_2_ fold-change; MaxFrac, fraction of contigs having the MaxDate timepoint.
