# Supplementary material for: Differential gene expression between viruliferous and non-viruliferous Schizaphis graminum (Rondani)
Source: PLoS One. 2023 Nov 8;18(11):e0294013. doi: 10.1371/journal.pone.0294013 (PMC10631655; doi:10.1371/journal.pone.0294013)
Supplement: S12 Table — (DOCX) [file pone.0294013.s013.docx]

| Cluster | Total | MinDate | MinFrac | MaxDate | MaxFrac |
| --- | --- | --- | --- | --- | --- |
| 1 | 6960 | 20 | 0.197 | 2 | 0.207 |
| 2 | 2165 | 5 | 0.398 | 15 | 0.358 |
| 3 | 2272 | 10 | 0.298 | 20 | 0.526 |
| 4 | 628 | 5 | 0.551 | 20 | 0.344 |
| 5 | 1050 | 5 | 0.328 | 20 | 0.359 |
| 6 | 106 | 3 | 0.387 | 0 | 0.566 |
| 7 | 441 | 10 | 0.338 | 15 | 0.413 |
| 8 | 211 | 5 | 0.720 | 0 | 0.479 |
| 9 | 208 | 0 | 0.615 | 3 | 0.438 |
| 10 | 680 | 2 | 0.394 | 20 | 0.340 |
| 11 | 110 | 0 | 0.318 | 5 | 0.473 |
| 12 | 290 | 15 | 0.479 | 5 | 0.610 |
| 13 | 476 | 5 | 0.527 | 20 | 0.464 |
| 14 | 218 | 5 | 0.711 | 20 | 0.381 |
| 15 | 234 | 20 | 0.329 | 3 | 0.295 |
| 16 | 104 | 0 | 0.433 | 15 | 0.529 |
| 17 | 222 | 0 | 0.527 | 3 | 0.342 |
| 18 | 303 | 10 | 0.248 | 5 | 0.620 |
| 19 | 46 | 20 | 0.630 | 5 | 0.696 |
| 20 | 87 | 2 | 0.460 | 0 | 0.402 |

Total, count of contigs in the cluster; MinDate, timepoint with the most minimum values of log_2_ fold-change; MinFrac, fraction of contigs having the MinDate timepoint; MaxDate, timepoint with the most maximum values of log_2_ fold-change; MaxFrac, fraction of contigs having the MaxDate timepoint.
