# Supplementary material for: Differential gene expression between viruliferous and non-viruliferous Schizaphis graminum (Rondani)
Source: PLoS One. 2023 Nov 8;18(11):e0294013. doi: 10.1371/journal.pone.0294013 (PMC10631655; doi:10.1371/journal.pone.0294013)
Supplement: S15 Table — (DOCX) [file pone.0294013.s016.docx]

| B-KSU | B-BH | H-KSU | H_BH | MED | MEAM | NAME |
| --- | --- | --- | --- | --- | --- | --- |
| up-regulated in efficient whitefly transmitter: | | | | | | |
| N | N | F | N | N | F | UDP-glucose4-epimerase |
| N | N | N | N | N | F | pyruvate kinase |
| N | N | N | N | N | F | GMP synthase |
| N | N | N | N | N | F | UTP-glucose1-phosphate uridyl transferase |
| N | N | N | N | N | F | NADPH cytochromeP450 reductase |
| N | N | N | N | N | F | phosphoglucosamine mutase |
| P | P | P | P | N | F | cathepsin F |
| N | N | N | N | N | F | chymotrypsin inhibitor-like |
| F | F | F | F | N | F | ATP-dependent RNA helicase |
| N | N | N | N | N | F | glutathione-S-transferase |
| N | N | N | N | N | F | viral A-type inclusion protein |
| N | N | N | P | N | F | zinc finger protein 596 |
| N | N | N | N | N | F | argininosuccinate lyase |
| N | N | N | N | F | F | eukaryotic translation initiation factor 3 subunit A |
| F | F | F | F | N | F | cathepsin B |
| N | N | N | N | N | F | activating signal co-integrator 1 complex subunit 2 homolog |
| N | N | N | N | N | F | 40S ribosomal protein S9 |
| N | N | N | N | N | F | ribosomal protein LP0 |
| P | P | N | P | F | N | phosphatidylethanolamine-binding protein |
| N | N | N | N | F | N | RUN and FYVE domain-containing protein 1 |
| N | N | N | N | F | N | mucin-2 isoform X2 |
| N | N | N | N | F | N | vesicle-associated membrane protein-like |
| N | F | N | F | F | N | glutathione peroxidase |
|  | | | | | | |
| down-regulated in efficient whitefly transmitter: | | | | | | |
| P | P | N | P | N | F | phosphatidylethanolamine-binding protein |
| P | P | P | P | N | F | cuticular protein analogous to peritrophin 3-B precursor |
| P | P | P | P | N | F | cuticular protein analogous to peritrophin 1-G |
| P | P | P | P | N | F | cuticle protein |
| P | P | P | P | N | F | endocuticle structural glycoprotein SgAbd-9 |
| P | P | P | P | N | F | RP1 cuticle protein 11 precursor |
| N | N | N | P | N | F | C-type lectin 5 |
| N | N | N | P | N | F | chondroitin proteoglycan 2 |
| N | N | N | N | N | F | putative defense protein Hdd11 |
| N | N | N | F | N | F | superoxide dismutase |
| P | P | P | P | N | F | myosin 8 |
| N | N | N | F | N | F | double stranded RNA-specific editase 2 |
| N | N | N | N | N | F | band4.1-like protein 3 |
| N | N | N | N | N | F | hydroxyacid-oxoacid transhydrogenase |
| P | P | N | N | N | F | acyl-coA dehydrogenase |
| F | F | N | N | N | F | 3-ketoacyl-coA thiolase |
| N | N | N | P | N | F | heat shock protein 70 |
| N | N | N | N | N | F | chaperone DNAK |
| N | N | N | N | N | F | 60kDa chaperonin 2 |
| N | N | N | N | F | N | protein disulfide isomerase |
| N | N | N | N | F | N | tropinone reductase 2-like isoform X1 |
| N | N | N | N | F | N | tubulin folding cofactor B |
| N | N | F | N | F | N | putative inorganic phosphate cotransporter |
| N | N | N | N | F | N | heat shock factor-binding protein 1-like |
| N | P | N | P | F | N | NADH dehydrogenase |
| N | N | N | N | F | N | nesprin 1 |
| F | N | F | N | F | N | catalase-like isoform X2 |
| N | N | P | N | F | N | succinate dehydrogenase flavoprotein subunit |
| N | N | N | N | F | N | methionine aminopeptidase 1 |
| F | N | N | N | F | N | muscle M-line assembly protein unc-89 isoform X2 |
| N | N | N | N | F | N | adenylate kinase 3 |
| N | F | N | N | F | N | 4-coumarate coA ligase 2 |
| N | P | P | N | F | N | cytochrome b-c1 complex subunit 2 |
| N | N | N | F | F | N | 28S ribosomal protein S15 |
| N | N | N | N | F | N | 28S ribosomal protein S22 |

Supplemental table 10. Comparison of most differential expression in *Bemisia tabaci* to differential expression in biotypes B and H. The 20 most up-regulated proteins and 20 most down-regulated proteins in each cryptic species of *B. tabaci* are compared to presence among the significantly differentially expressed contigs in *S. graminum* biotypes B and H at false-discovery rate = 0.05. Column headings: B-KSU, biotype B with KSU transcriptome; B-BH, biotype b with BH transcriptome; H-KSU, biotype H with KSU transcriptome; H-BH, biotype H with BH transcriptome; MED, Mediterranean cryptic species in *B. tabaci s.l.;* MEAM, Middle Eastern-Asia Minor cryptic species in *B. tabaci s.l.* Values: F, found; N, not found; P, possibly found. Data for MED, MEAM, and NAME come from Figures 2 and 3 in Kliot et al. (2020).
